# Local origin or external input: modern horse origin in East Asia

**DOI:** 10.1186/s12862-019-1532-y

**Published:** 2019-11-27

**Authors:** Tiao Ning, Yinghui Ling, Shaoji Hu, Arman Ardalan, Jing Li, Bikash Mitra, Tapas Kumar Chaudhuri, Weijun Guan, Qianjun Zhao, Yuehui Ma, Peter Savolainen, Yaping Zhang

**Affiliations:** 1 College of Agriculture, Kunming University, Kunming, 650214 Yunnan China; 2 Laboratory for Conservation and Utilization of Bio-resource and Key Laboratory for Microbial Resources of the Ministry of Education, Yunnan University, Kunming, 650091 Yunnan China; 3 Institute of Animal Science, Chinese Academy of Agricultural Sciences, Beijing, 100193 China; 4 College of Animal Science and Technology, Anhui Agricultural University, Hefei, 230036 Anhui China; 5 Institute of International Rivers and Eco-security, Yunnan University, Kunming, 650214 Yunnan China; 6 Department of Gene Technology, Science for Life Laboratory, KTH Royal Institute of Technology, SE-171 65, Solna, Sweden; 7 The Research Center for Urban Modern Agricultural Engineering of Yunnan Tertiary Education, Kunming University, Kunming, 650214 Yunnan China; 8 Department of Zoology, University of North Bengal, Cellular Immunology Laboratory, Siliguri, West Bengal 734013 India; 9 State Key Laboratory of Genetic Resources and Evolution Kunming, Yunnan, Kunming Institute of Zoology, Chinese Academy of Sciences, Wuhua, 650223 Yunnan China

**Keywords:** Local origin, External input, Domestic horse, East Asia

## Abstract

**Background:**

Despite decades of research, the horse domestication scenario in East Asia remains poorly understood.

**Results:**

The study identified 16 haplogroups with fine-scale phylogenetic resolution using mitochondrial genomes of 317 horse samples. The time to the most recent common ancestor of the 16 haplogroups ranges from [0.8–3.1] thousand years ago (KYA) to [7.9–27.1] KYA. With combined analyses of the mitochondrial control region for 35 extant Przewalski’s horses, 3544 modern and 203 ancient horses across the world, researchers provide evidence for that East Asian prevalent haplogroups Q and R were indigenously domesticated or they were involved in numerous distinct genetic components from wild horses in the southern part of East Asia. These events of haplotypes Q and R occurred during 4.7 to 16.3 KYA and 2.1 to 11.5 KYA, respectively. The diffusion of preponderant European haplogroups L from west to East Asia is consistent with the external gene input. Furthermore, genetic differences were detected between northern East Asia and southern East Asia cohorts by Principal Component Analysis, Analysis of Molecular Variance test, the χ^2^ test and phylogeographic analyses.

**Conclusions:**

All results suggest a complex picture of horse domestication, as well as geographic pattern in East Asia. Both local origin and external input occurred in East Asia horse populations. And besides, there are at least two different domestication or hybridization centers in East Asia.

## Background

The role of the domestic horse (*Equus caballus*) was quite different from that of other domestic animals. The horse domestication was believed to develop greatly the mobile pastoralism and ancient overland trade routes, which contributed to the ancient civilization to flourish [1]. In other words, the domestic horse demography was intensively influenced by humans. A former study has revealed that both signatures of recent positive selection and recent population expansion were detected on mtDNA of domestic horse [2]. The scenarios of horse domestication provide an extensive comprehension of human history. Some researches indicate that the western part of the Eurasian steppe and the Iberian Peninsula are considered as the horse’s domestication regions [3–7]. However, the domestication scenario of horse remains unclear. One reason is that female lineages of wild horses have been introduced into the domestic horse population through domestication and hybridization in several regions across Eurasia [8–10]. Przewalski’s horse is the only remaining wild horse. Recent researches indicate that Przewalski’s horse and domesticated horse diverged about 45,000 years ago and have remained connected gene flow [11]. However, Przewalski’s horse is not an ancestor of modern domestic horses but the feral descendant of the domesticated Botai horse [12]. The wild ancestor of domestic horses seems to be extinct presently [13]. The other reason is that the identification of horse domestication history has been problematic without a clear domestication scenario of the horse in East Asia. Multiple maternal origins and high genetic diversity have been characterized in East Asia using a small number of mtDNA D-loop, mitochondrial gene and microsatellite data [14–18]. The domestication history of the horse in East Asia remains unanswered in detail.

China and Mongolia are two famous horse-breeding countries. The current number of domestic horses, according to FAOSTAT Database 2009, is about 67 million (the most in any country) and 22 million in China and Mongolia, respectively. Besides, East Asia holds important eco-regions of the Steppe, such as Daurian forest, Emin Valley, Mongolian-Manchurian grassland and Tian Shan foothill arid steppe. According to literature and archaeology records, the nature of domestic horse origins in East Asia is explained by two alternative hypotheses: the external input hypothesis and the local origin hypothesis. The external input hypothesis suggests that domestic horses in East Asia were introduced of an outside geographic area, possibly from the northern Llano of Black and Caspian seas, West Asia or even Europe by exchange [19]. Evidence for this hypothesis resulted from the domesticated horses (partly for identification trouble) that were not discovered in the early Shang Dynasty (3600BP) but was presented with a carriage during the late Shang (3300BP) [20]. Under the external hypothesis, the genetic components of domestic horses in East Asia should be a subset derived from the West part of Eurasia. The local origin hypothesis proposes that domestic horses in East Asia were indigenously tamed by local ethnic people [21–23]. Several wild horse species were widely distributed across East Asia during the Pleistocene [24, 25], leaving open the possibility of the indigenous domestication within East Asia. In this origin scenario, horses in East Asia were supposed to harbor unique genetic components. Also ancestral haplotypes should have a moderate frequency in East Asia horse populations but are rare or absent in other population.

Based on the first hypervariable region (HVR1) sequences, the fine phylogeny cannot be resolved. Complete mitochondrial DNA sequences have proved to be very useful in reconstructing the past for human and livestock [26–29], considering that all available data could be dissected into respective haplogroups and insight into special matrilineal phylogeographic structure depending on the high resolved mitochondrial genome phylogeny. Although the latest study has generated a consensus tree using 66 full horse mtDNA sequences [30], the tree did not show all haplogroups. Meanwhile, this work has not performed a detail genetic comparison at a lineage level for horse populations of the world. In this study, researchers attempt to discriminate between the local origin and the external input hypotheses, reconstruct the past of horses in East Asia, and determine the resolution of horse phylogeny and phylogeography by analyzing both complete mitochondrial genome and large-scale HVR1 sequences.

## Methods

### Sampling protocol

A total of 28 Przewalski’s wild horses from Xinjiang Province of China, and 1554 modern domestic horses from Europe, Iran, India, and China were collected and sequenced for a 385 bp fragment of the mtDNA control region (CR). The process of collecting horse samples is strictly based on the animal protection laws of the countries mentioned above. The following collection method was used for sampling: 1) prick the veins in the neck of the horse with a sterile needle, then absorb the blood sample with a medical blood collection card, and finally press the wound with a sterile cotton ball to stop the bleeding. 2) gently pull five manes with hair follicles from each horse’s bristles. 3) use medical cotton swabs to gently wipe the oral wall of the horse and apply the cotton swabs to the buccal swabs. Then, 93 samples covering the mtDNA diversity for the major horse haplogroups identified of preliminary minimum spanning network analysis on the 385 bp (15,450–15,834 NPS) dataset were selected for sequencing the near-complete mitochondrial genome (excluding repeat regions in CR) (Additional file 12: Figure S1). All novel sequences have been submitted to GenBank (Additional file 1: Table S1). We incorporate the maximum number of modern and ancient horse sequences from GenBank. A truncated 247 bp segment (15,494–15,740 NPS) of the CR were analyzed (Additional file 2: Table S2). Additionally, the NPS 15,494–15,740 is convenient for comparison and discussion as they were widely used in previous horse studies [5, 10]. The final 247 bp dataset comprised 41 extant Przewalski’s wild (28 novel and 13 GenBank), 3554 modern (1554 novel and 1848 GenBank) and 203 ancient (154 ancient domestic, 12 ancients but no time record and 37 extinct wild) horses across the world. All samples were divided into relative geographic populations. The definitions of geographic regions were shown in Additional file 3: Table S3. Besides, 217 available modern and seven extant wild (*Equus przewalskii*) horses GenBank entries (EF597512, AY584828, and EU939445 were disregarded as the same reason mentioned in [30] were combined with 93 novel mtDNA genomes to obtain phylogenetic resolution.

### PCR amplification and sequencing

Genomic DNA was extracted from blood, tissue, hair or buccal swabs. Primer pairs HDF and HDR were used to amplify the 385 bp fragment of the control region for DNA template from blood, tissue, and hair [31]. Nest PCR was performed for DNA from buccal swabs. The near-complete mtDNA sequences were amplified by four pairs of primers, and then each fragment was sequenced using a set of primers (Additional file 4: Table S4). All sequences were aligned with both forward and reverse direction by DNASTAR 5 (DNAstar Inc. Madison, Wisconsin, USA).

### Data analyses

#### Phylogeny tree

**Fig. 1:**
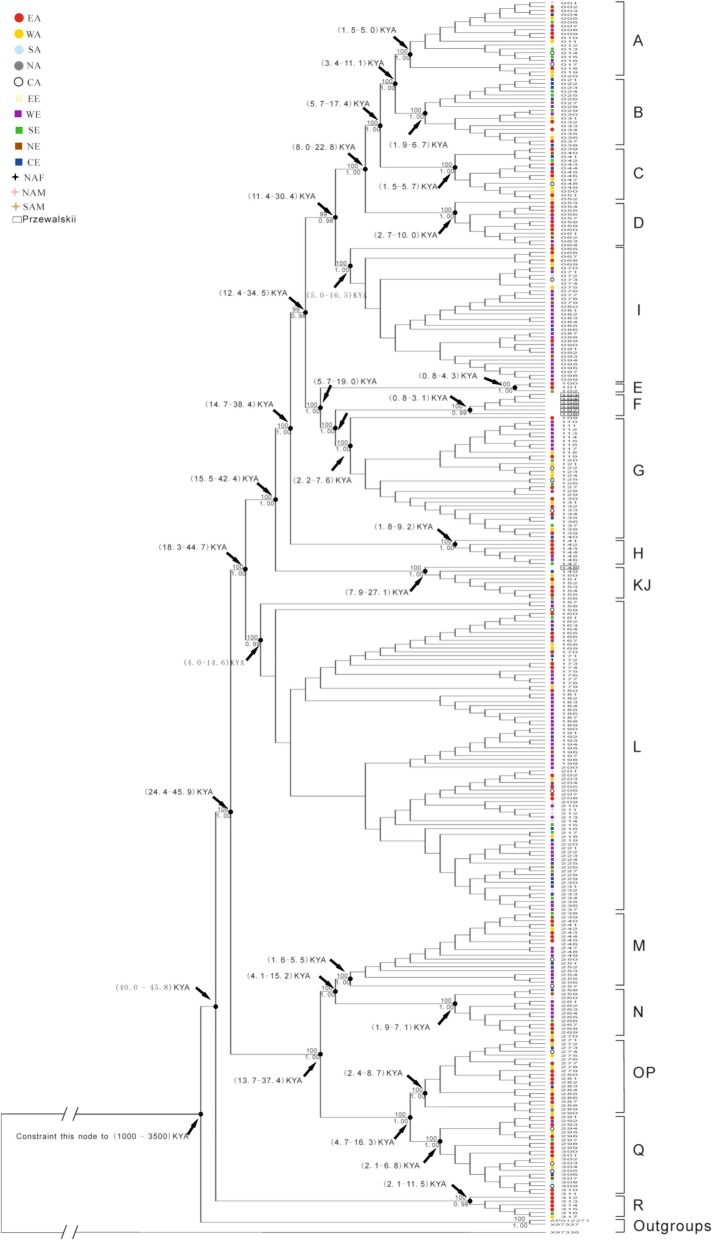
The phylogeny tree constructed from mitochondrial genome sequences. Note: The numbers above the branches are the Bayesian posterior probabilities and the numbers below the branches are the bootstrap proportions derived from the parsimony analysis. For the Bayesian analysis, the best substitute model GTR + I + G was used, and 20 million generations were executed. One sampled tree from the last 20,000 generations, which matched the tree with all clades/haplogroups, was used for the Bayesian MCMC by BEAST. The time to a most recent common ancestor (TMRCA) for clades/haplogroups was marked along with the node

Bayesian inference tree was constructed with MrBayes 3.1.2 [32] using the best-fit model (GTR + I + G: shape а = 0.4890; pinvar = 0.7620) identified by jModeltest 0.1.1 [33]. Three independent runs were performed, each run with 20 million generations. Besides, maximum parsimony (MP) trees were constructed by using a heuristic search with 100 random addition sequences and tree-bisection-reconnection (TBR) branch swapping option in PAUP 4.0b10 [34]. Robustness of the MP tree nodes was estimated by bootstrapping with 1000 replications. Trees were rooted using domestic donkey mtDNA sequences (*Equus asinus*, X97337 and AP012271) and a Rhinos mtDNA sequence (*Rhinoceros unicornis*, X97336). The posterior probabilities and bootstrap proportions were listed on the tree (Fig. 1).

#### Haplogroup classification

Specific mutation motifs (characteristic mutations exclusively shared by its members) of each haplogroup were inferred from the phylogenetic tree. The phylogenetic tree only constructed on 93 near complete modern horse mtDNA genome sequences. The topology of the consensus tree based on the 93 modern sequences is similar to that of the consensus tree based on the latest published 224 modern horse sequences. And a hierarchical haplogroup nomination system was used. They are both strategies described in anthropologic researches [35–37]. All sequences of the 247 bp (15,494–15,740 NPS) dataset were assigned to respective haplogroups by haplogroup-specific motif and (near-) matching strategy, based on the classification backbone (Additional file 13: Figure S2). Since mutation motifs are not found between 15,494–15,740 NPS for haplogroup A, B, C, G, K, and J, researchers did not detailedly analyze and discuss them in this study. To justify the inferred haplogroups, samples having enough templates were selected to further screen-specific mutations on some coding regions. All novel sequences of coding regions have been submitted to GenBank (Additional file 1: Table S1). Extensive sampling and coding regions sequencing will help to discuss the scenario of haplogroups A, B, C, G, K, and J.

#### Haplotype networks

The truncated 247 bp (15,494–15,740 NPS) dataset of the CR containing the maximum number of novel and GenBank horse sequences (including modern and ancient samples) was adopted to create and visualize haplotype networks using HapStar-0.7 [38]. HapStar generates the network graph as a minimum spanning tree between all haplotypes, that is, computing the lowest total Hamming distance (number of mismatches) between all haplotypes.

#### Estimation of time to a most recent common ancestor (TMRCA)

A Bayesian tree of near-complete mtDNA genomes was used for estimating the substitution rate and the TMRCA for each significant haplogroups. The analysis was implemented by using the Bayesian Markov Chain Monte Carlo (BMCMC) in the BEAST1.6 [39]. The fossil record for *Equus* was accepted to be the time region 1–3.5 million years ago [10, 40, 41] as calibration to the separation time of horse and donkey. Bayesian Evolutionary Analysis Sampling Trees (BEAST) analyses were also run twice for six model frameworks. The six models are strict clock rate and constant population size (SC-C), tight clock rate and exponential population size (SC-E), strict clock rate and population size estimated from Bayesian skyline plot (SC-S), strict clock rate and expansion population size (SC-EXPA), relaxed exponential clock rate and population size determined from Bayesian skyline plot (REC-S) and relaxed lognormal clock rate and population size estimated from Bayesian skyline plot (RLGC-S). The estimated sample size (ESS) values of all parameters were higher than 200, which were considered to be a sufficient level of sampling. A Bayes factor analysis was used to judge the optimization model for BEAST estimation [39, 42].

#### Population genetic analyses

The population genetic parameters and the Analysis of Molecular Variance (AMOVA) were calculated with ARLEQUIN3.5 software [43]. Principal component analysis (PCA) and the Pearson χ2 test were conducted using haplogroup frequencies by SPSS13.0 (SPSS, Inc., Chicago, IL). Contour maps based on haplogroup frequencies and principal components were drawn by Surfer 8.0 (Golden Sofware, Inc).

## Results

### Phylogeny and Haplogroup profile

The general topology of the consensus Bayesian tree is similar to that of maximum parsimony tree, drawn using 317 near-complete mitochondrial genome sequences (Fig. 1). The fine phylogeny resolution was presented with highly supporting Bayesian posterior probabilities (≥ 0.98) and bootstrap proportions (≥ 99%) for 16 haplogroups. The consensus topology was slightly different from the previously defined distinct clades and haplogroups based on HVR1 [9] and mitochondrial genome data [44] (Additional file 5: Table S5), respectively: because the modified hierarchical haplogroup nomination system was used for convenient systematical analysis. In total, 16 haplogroups were identified in this study (Fig. 1). The four independent haplogroups J, K, O, and P identified by a previous study [44] were assorted into haplogroup KJ and haplogroup OP, respectively, according to the bootstrap proportion and the Bayesian posterior for haplogroups node in the study. Moreover, it is noteworthy that the apparent divergence groups were revealed by phylogenetic analysis, which indicates that horse mtDNA sequences fall into two very distinct groups. One contains haplogroup R, and the other provides the remaining 15 haplogroups.

### TMRCAs

The time to most recent common ancestors for all haplogroups was estimated using Bayesian Markov Chain Monte Carlo (MCMC) searches with six model frameworks: SC-C, SC-E, SC-S, SC-EXPA, REC-S, and RLGC-S. The REC-S framework could not be estimated reliably since the estimated sample size (ESS) for the node age of haplogroups were not larger than 200, even when researchers run 350,000,000 MCMC steps. Since the RLGC-S model was substantially better than the SC-C, the SC-E, the SC-S and the SC-EXPA with Bayes factor difference of the means of the tree likelihoods greater than six [39, 42], the RLGC-S model is appropriate for the tMRCAs estimation of domestic horses. The BMCMC estimation of each haplogroup by the RLGC-S model running 200,000,000 MCMC steps was showed in (Additional file 6: Table S6). Calibration age 1000 KYA and 3500 KYA, were assumed as the minimum and maximum fossil benchmark for *Equus* [10]. The minimum was the latest date for the development of one cranial character common to all extant equid species [41], while the maximum is the generally accepted age for the earliest known *Equus* fossil, *Equus simplicidens* [40]. The tMRCAs for all haplogroups were diverse on the consensus tree (Fig. 1). For instance, the tMRCA of haplogroup F is the youngest (0.8–3.1 KYA), while the tMRCA of haplogroup KJ is the oldest (7.9–27.1 KYA). Besides, the tMCRAs of modern domestic horse mtDNA haplotypes was between 40.0 to 45.8 KYA ago, indicating a much early divergence between haplogroup R and the other horse haplogroups.

### Principal component analysis

**Fig. 2:**
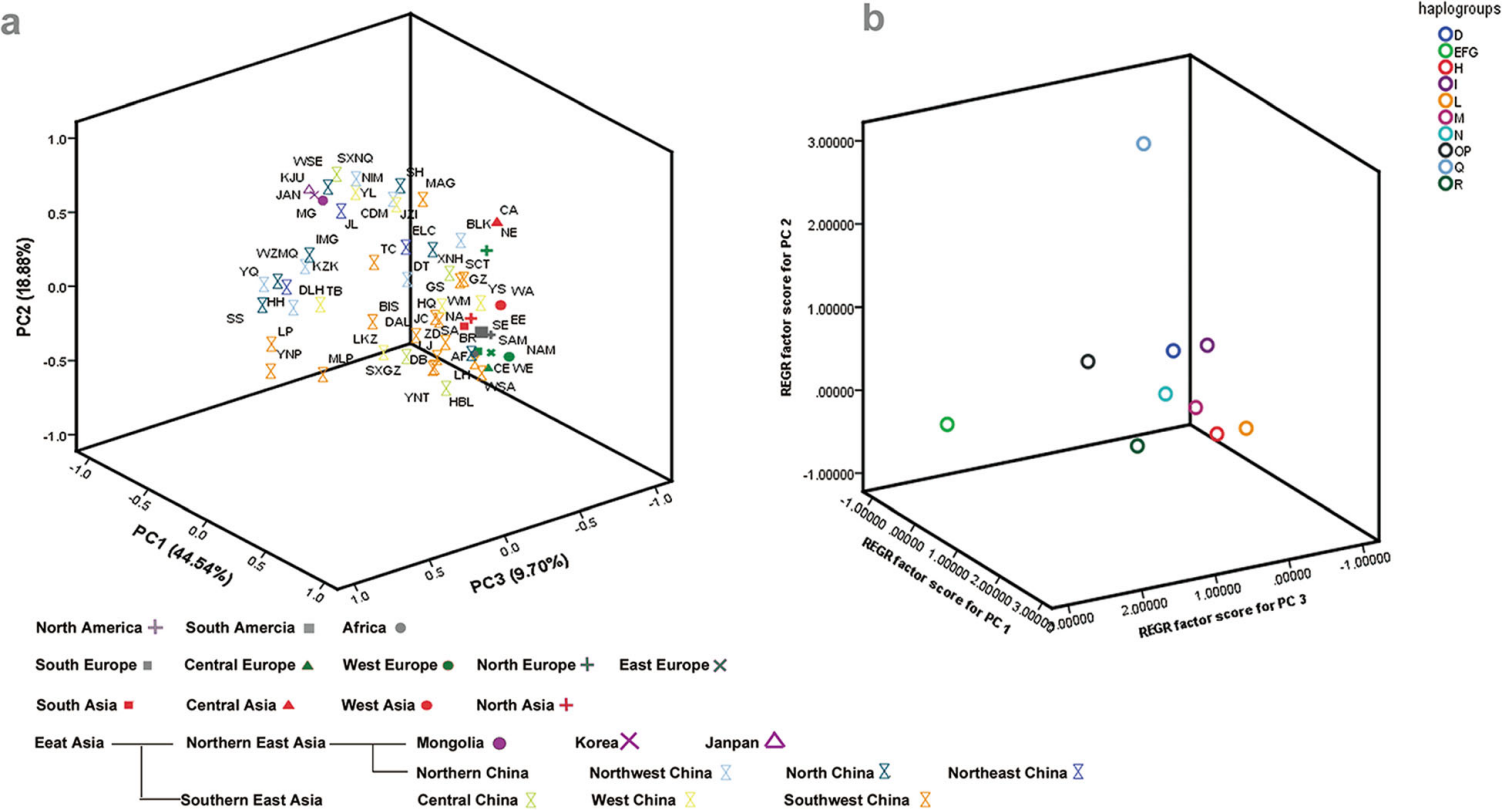
Three dimensional PCA of populations analyzed in the present study. **a** PC map of the 59 world horse populations based on haplogroup frequencies, for more details, see Table 1. **b** Plot of the haplogroup contribution of the first, second and third PCs. The contribution of each haplogroup was calculated as the factor scores for PC1, PC2 and PC3 with regression (REGR) method in SPSS

**Fig. 3:**
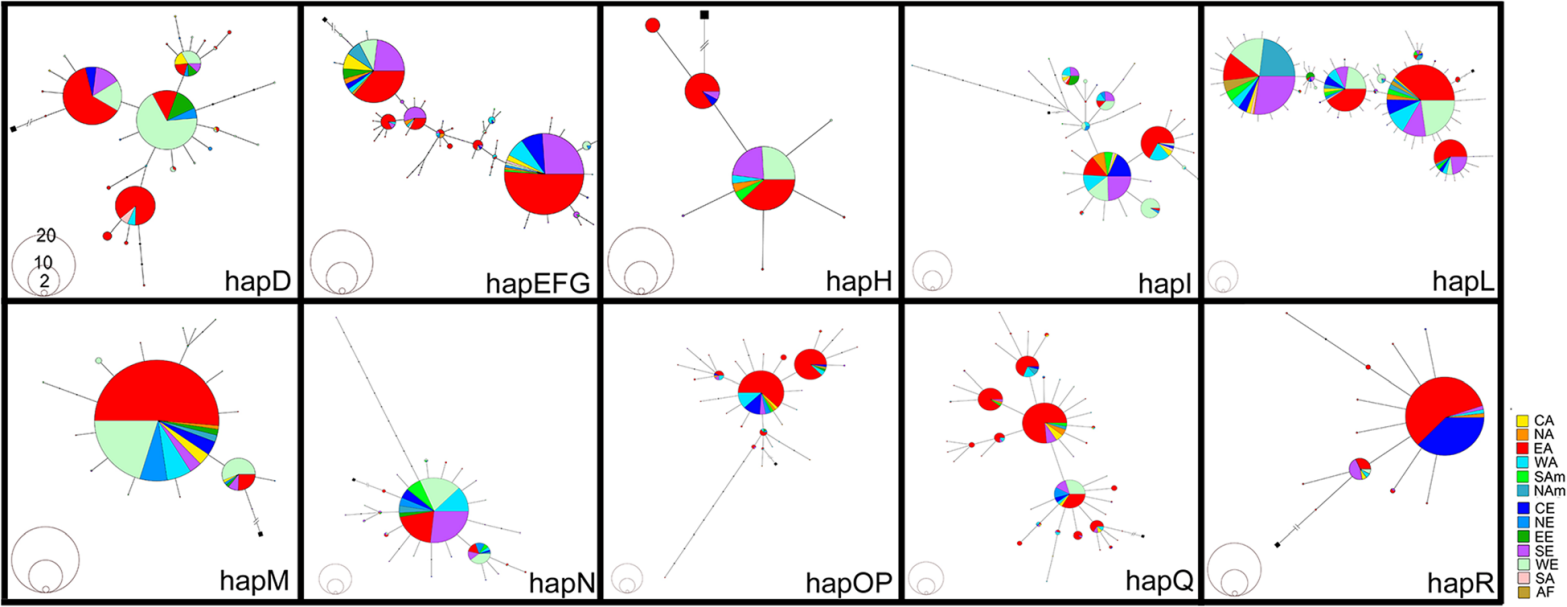
Minimum spanning network of horse haplogroups. The smallest frequency circle to the biggest circle is 2, 10 and 20, respectively.

**Fig. 4:**
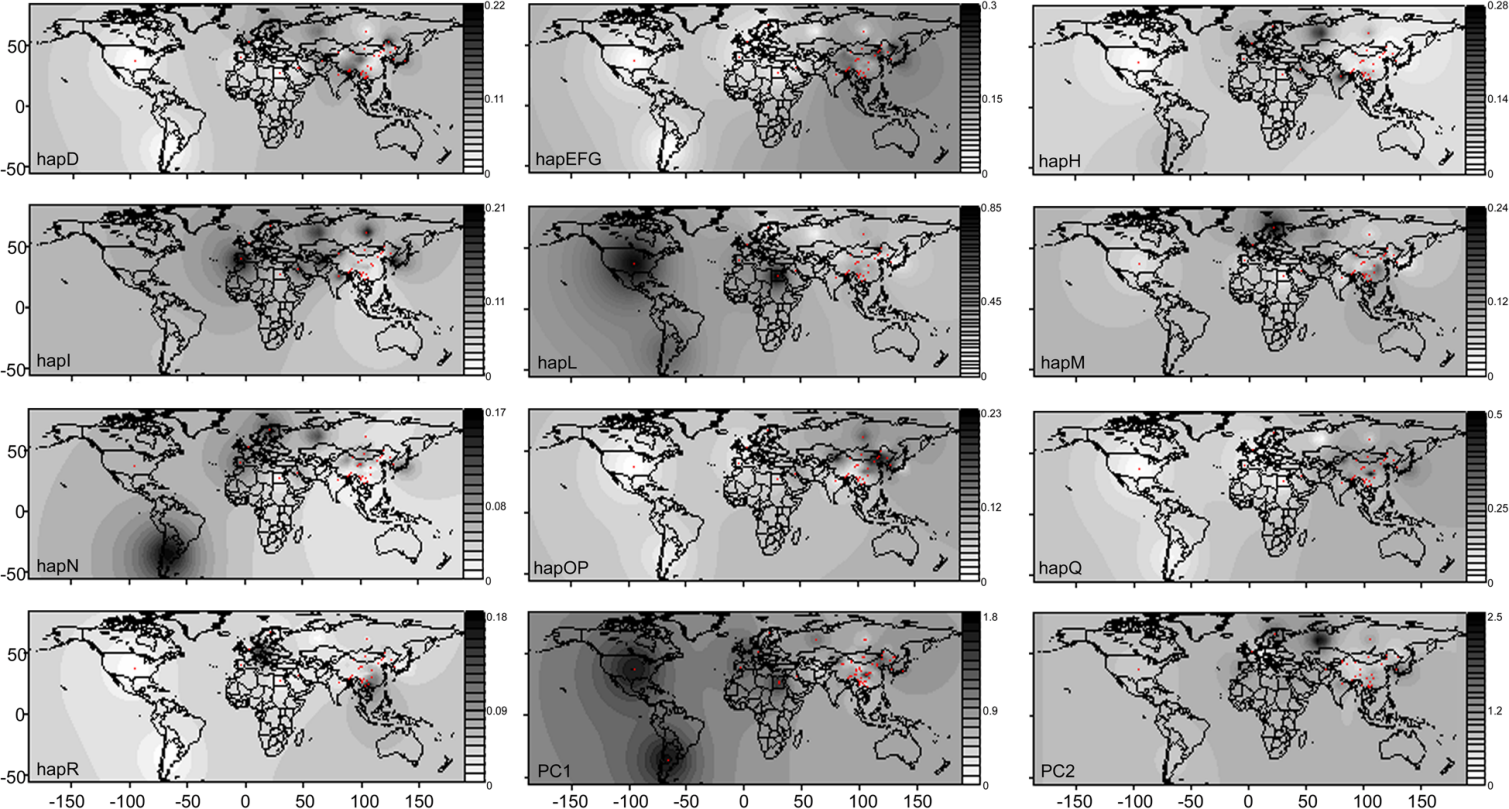
Contour maps of haplogroup frequencies in geographical populations and the first two principal components. Note: We took the outline map from the national basic geographic information center (http://www.ngcc.cn) as the base map, and then drew it ourselves

Based on horse mtDNA HVR1 haplogroup frequencies (Table 1), population relationship was investigated among 59 horse populations by principal analysis (Fig. 2). The first three and the primary two principal components account for 73.12 and 63.42% of the total variation, respectively. The first PC (44.54%) is approximately east-west. The central Asian horse population is located between east and west (Fig. 2a). This pattern is consistent with geographical distribution. Meanwhile, it reveals the importance of Central Asia in east-west communication of horse populations. The main haplogroups contributing to the first PC is EFG concentrated at the east pole; the haplogroups H, I, L, M and N are concentrated at the west pole (Fig. 2b). Haplogroup EFG is the most frequent haplogroup in both the east and the west but occurs at frequencies of about 30% in the west, whereas the frequency is generally about 50% in the east. Haplogroups I, L and N are predominantly in the west. Haplogroup H is frequent in both the east and the west but more prevalent in the west than in the east (Table 1, Table 2, Fig. 3, Fig. 4, Additional file 9: Table S9 and Additional file 15: Figure S4). The second PC (18.88%) is primarily north-south, separating northern East Asia (NEA) horse populations from southern East Asia (SEA) horse populations. The NEA horse populations relatively alone gather together as compared with the SEA. Most of the SEA horse populations cluster together with West Asia (WA), South Asia (SA), North Asia (NA), Central Europe (CE), West Europe (WE) and South Europe (SE) horse populations. This suggests genetic divergence between the SEA and the NEA and their geographical pattern. The main haplogroups contributing to the second PC is that I, N, OP and Q are relatively concentrated at the north pole; the haplogroups H, L, M, an R are concentrated at the south pole (Fig.2b). This suggests the different haplogroup distribution between the NEA horse population and the SEA horse population (Table 1, Table 2, Fig. 4, Additional file 14: Figure S3 and Additional file 15: Figure S4). To reveal this difference in detail, we subdivide the East Asian population data step by step for making principal component analysis (Additional file 16: Figure S5). The results show that the frequency of haplotype distribution is different between the NEA horse population and the SEA horse population. These imply that their domestication backgrounds are different.

**Table 1:** The frequency distribution of modern horse mtDNA control-region haplogroups across the world

		breed, region, country	sample size	haplogroups									
				D	I	EFG	H	L	M	N	OP	Q	R
NEA	1	YL, NWC, China	13	0	15.4	7.7	0	15.4	0	0	23.0	38.5	0
	2	KZK, NWC, China	43	4.7	2.3	20.9	2.3	16.3	9.3	11.6	4.7	20.9	7.0
	3	YQ, NWC, China	22	4.5	9.2	22.7	4.5	13.6	4.5	9.2	18.2	13.6	0
	4	BLK, NWC, China	50	8.0	2.0	2.0	6.0	30.0	14.0	4.0	8.0	26.0	0
	5	CDM, NWC, China	24	20.8	0	8.3	0	25.0	0	4.2	4.2	37.5	0
	6	DT, NWC, China	30	16.7	3.3	13.3	6.7	23.3	3.4	13.3	0	16.7	3.3
	7	DLH, NWC, China	13	15.4	7.7	38.5	0	23.0	0	0	0	15.4	0
	8	MG, MG, Mongolia	50	0	8.0	14.0	0	14.0	6.0	12.0	16.0	28.0	2.0
	9	IMG, NC, China	76	1.3	5.3	18.4	1.3	19.7	3.9	6.6	21.1	21.1	1.3
	10	WSE, NC, China	12	0	0	16.7	0	16.7	0	0	16.7	50.0	0
	11	SS, NC, China	13	0	0	30.7	0	15.4	15.4	0	23.1	15.4	0
	12	WZMQ, NC, China	15	0	0	26.7	0	20.0	0	0	33.3	20.0	0
	13	XNH, NC, China	30	3.3	10.0	3.3	0	26.7	6.7	6.7	16.6	20.0	6.7
	14	BR, NC, China	1	0	0	0	0	100.0	0	0	0	0	0
	15	SH, NC, China	12	8.3	16.7	8.3	0	16.7	8.3	0	0	33.4	8.3
	16	ELC, NEC, China	29	24.1	3.4	10.4	0	24.1	7.0	3.4	3.4	20.8	3.4
	17	HH, NEC, China	17	0	11.8	29.4	0	17.6	11.8	0	11.8	17.6	0
	18	JL, NEC, China	25	12.0	8.0	8.0	8.0	12.0	0	4.0	24.0	20.0	4.0
	19	KJU, FE, Korea	25	12.0	24.0	12.0	4.0	0	0	8.0	20.0	20.0	0
	20	JAN, FE, Japan	9	11.1	11.1	22.2	0	11.1	0	11.1	0	33.4	0
SEA	21	GS, CC, China	6	0	0	0	0	50.0	0	0	0	33.3	16.7
	22	SXNQ, CC, China	14	0	7.1	14.3	0	14.3	0	0	14.3	50.0	0
	23	SXGZ, CC, China	70	0	0	8.5	0	34.3	28.6	0	28.6	0	0
	24	HBL, CC, China	14	0	0	7.1	0	35.8	28.6	7.1	0	0	21.4
	25	JZI, WC, China	17	11.8	5.9	11.8	0	23.5	11.8	0	0	35.2	0
	26	LKZ, WC, China	10	0	0	30.0	0	50.0	10.0	0	0	10.0	0
	27	NIM, WC, China	13	30.8	0	7.7	0	7.7	7.7	0	7.7	30.8	7.7
	28	TB, WC, China	18	22.2	0	33.3	0	27.8	0	0	0	16.7	0
	29	YS, WC, China	7	0	28.6	0	0	57.1	0	0	0	14.3	0
	30	HQ, WC, China	33	6.0	3.0	9.1	3.0	42.4	6.1	0.0	6.1	18.2	6.1
	31	SCT, SWC, China	3	0	0	0	0	66.7	0	0	0	33.3	0
	32	YNT, SWC, China	6	0	0	16.7	0	66.6	0	16.7	0	0	0
	33	ZD, SWC, China	22	9.1	4.6	13.6	9.1	31.8	22.7	0	0	9.1	0
	34	LJ, SWC, China	16	25.0	6.3	12.5	12.5	37.4	0	0	6.3	0	0
	35	LH, SWC, China	4	0	0	0	50.0	25.0	25.0	0	0	0	0
	36	JC, SWC, China	33	9.1	3.0	6.1	6.1	24.1	18.2	6.1	18.2	9.1	0
	37	TC, SWC, China	17	0	0	17.6	0	35.3	5.9	0	11.8	29.4	0
	38	DAL, SWC, China	23	8.7	0	13.0	0	34.9	13.0	0	4.4	13.0	13.0
	39	WM, SWC, China	17	5.9	0	11.8	0	52.9	5.8	0	0	17.7	5.9
	40	GZ, SWC, China	73	6.8	6.8	5.6	8.2	32.9	6.8	0	4.1	20.6	8.2
	41	LP, SWC, China	12	8.3	0	41.7	33.4	0	0	0	0	8.3	8.3
	42	DB, SWC, China	19	0	0	15.8	0	52.6	15.8	0	0	5.3	10.5
	43	WSA, SWC, China	42	4.8	2.4	2.4	14.3	38.0	9.5	0	7.1	4.8	16.7
	44	YNP, SWC, China	16	0	0	62.5	0	31.2	0	0	6.3	0	0
	45	MAG, SWC, China	4	0	0	0	0	25.0	25.0	0	0	50.0	0
	46	MLP, SWC, China	8	0	0	12.5	0	12.5	12.5	0	25.0	0	37.5
	47	BIS, SWC, China	22	18.2	0	13.6	4.6	18.2	9.1	0	0	13.6	22.7
SA	48		12	8.3	16.7	8.3	33.3	25.0	0	0	0	8.3	0
CA	49		48	12.5	18.8	0	8.3	20.8	14.6	0	2.1	20.8	2.1
NA	50		38	0	21.1	2.6	10.5	34.2	2.6	2.6	15.8	7.9	2.6
WA	51		170	1.2	14.1	0.6	13.5	33.5	6.5	7.1	10.0	12.4	1.2
EE	52		23	13.0	17.4	0	26.1	8.7	13.0	13.0	8.7	0	0
SE	53		366	1.1	20.5	2.5	7.1	44.5	3.0	10.7	2.5	6.0	2.2
CE	54		129	0.8	10.9	0.8	9.3	40.3	4.7	7.0	7.0	3.1	16.3
NE	55		51	11.8	9.8	0	9.8	11.8	23.5	13.7	5.9	13.7	0
WE	56		436	11.0	6.9	2.1	14	40.8	13.5	8.3	0	3.2	0.2
AF	57		25	0	4.0	0	4.0	88.0	0	0	4.0	0	0
NAM	58		86	0	7.0	0	0	82.6	3.5	5.8	0	1.2	0
SAM	59		54	0	5.6	0	9.3	55.6	7.4	16.7	1.9	3.7	0

Note: abbreviation used for populations are consistent with Additional file 3: Table S3

**Table 2:** The *P* value of Pearson χ^2^ test

Haplogroup	Cohort (SEA vs. NEA)	Cohort (E + CWA vs. E)
D vs. others	3.27E-01	9.34E-01
EFG vs. others	6.00E-04	1.60E-01
H vs. others	2.69E-02	3.04E-02
I vs. others	6.11E-05	3.96E-12
L vs. others	1.73E-01	3.80E-17
M vs. others	8.10E-03	1.28E-01
N vs. others	5.73E-26	6.35E-10
OP vs. others	1.13E-15	1.73E-08
Q vs. others	5.42E-12	5.15E-12
R vs. others	9.69E-02	2.12E-01

Note: cohort abbreviation used for populations are consistent with Table 1 and Additional file 3: Table S3. the northern East Asia (NEA), the southern East Asia (SEA), Europe (E), central west Asia (CWA)

### Analysis of molecular variance (AMOVA) and the χ^2^ test

To test the difference between the NEA and the SEA, we performed the AMOVA analysis and the χ^2^ test. It was indicated by the AMOVA result that the variation between the NEA and the SEA is 1.35 but significant (*P* = 0.036), although most change comes from within populations in East Asia. Moreover, according to the χ^2^ test, there is a significant difference (*P* < 0.05) between the SEA population and the NEA population belonging to the following seven haplogroups (EFG, H, I, M, N, OP, and R) (Table 2).

### Gene flow

Gene flow into or out of a population may be responsible for a marked change in allele frequencies. From the result of the degree of gene flow among horse populations (Additional file 7: Table S7), it can be observed that the gene flow between populations from the NE and the NEA, between populations from the NE and the EE and between populations from the NE and the SAM approached infinity, while the gene flow between the remaining populations varied from 1.4367 to 186.182. Much higher levels of gene flow were detected between the NEA and the SAM populations, between the NEA and the WA populations and between the NA and the SAM populations. Interestingly, the gene flow between the NEA and others are very different from that between the SEA and others. The NEA populations frequently flow with the west populations (the WA, the WE, and America), the north populations (the NA and the NE) and East Europe. However, the SEA populations frequently flow with the east population (the AF), the central populations (the CA and the CE) and the south populations (the SA and the SE).

### Phylogeographic analysis

Minimum spanning network was drawn to show the phylogeographic structure of modern horse haplogroups (Fig. 3). Principal components contour maps which map haplogroup frequency or principal component value to geographic coordinates were shown in Fig. 4. Meanwhile, the distribution of the dominant haplotypes of haplogroups was performed in horse populations (Additional file 8: Table S8). One hundred forty-four samples were classified into haplogroup D. The ancestral haplotypes of the haplogroup D are distributed in East Asia but are prevalent in East and West Europe now. The combined haplogroups E, F and G (haplogroup EFG) widely occur in the world. Samples of its ancestral haplotype come from West Asia. The EFG observed in northern East Asia show high frequencies. The ancestral haplotype of the haplogroup H distributes in East Asia, Central Europe, and South Europe. The haplogroup I is popular in West Europe and South Europe. Haplogroup L is the biggest one of the analyzable haplogroups in this study, which holds 884 samples. The source of an ancestral haplotype of haplogroup L is from West and South Europe. North America and Africa are dense with high frequency in haplogroup L on the contour map. Haplogroup M, with the sample size 208, is prevalent in Europe and Central-West Asia. Herein, 63 and 28 samples belong to southern East Asia and northern East Asia in haplogroup M, respectively. Interestingly, ancestral haplotype of haplogroup N presents in East Asia, however, none of 61 Central Asia horse was found in this haplogroups, and just 3 of 923 SEA samples are observed in haplogroup N although it is popular in West Asia, Southern Europe, North Europe, West Europe, South America and East Asia (especially the NEA in Additional file 14: Figure S3). Both the ancestral haplotype of haplogroup OP and haplogroup Q are prevalent in East Asia (also particularly in the NEA in Additional file 14: Figure S3). Although the total sample size of the NEA (718) is smaller than that of the SEA (923), samples belonging to the NEA (67) is more significant than that of the SEA (44). The last haplogroup R is prevalent in Central Europe and East Asia, while Southern East Asia is dense with high frequency in this haplogroup on the contour map (Fig. 4). Therefore, it can be noted that most of the haplogroups occur in the NEA and the SEA shows different distributions.

## Discussion

The origin of domestic horse in East Asia has long been a puzzle to archaeologists. According to history and archaeology records, two controversial hypotheses have been proposed about the origin of domestic horse in East Asia: the external input and the local origin. Multiple lines of evidence from history, archaeology and genetics on large-scale sampling, should enable us to understand the scenario of horse domestication in East Asia better.

### Testing the external input hypothesis

Archaeologists suggested the external input hypothesis of domestic horses in East Asia since the appearance time of domesticated horse in East Asia seems to be later than that concerning Central Asia. The earliest undisputed domestic horse was found in Kazakhstan, dating to about 5500 BP [4]. Domestic horse in China did not widely appear at archaeological sites before the Late Shang Dynasty (3300–3046 BP) in the middle and lower Yellow River regions [45]. Horse remains excavated in Korea and Japan was even later than that in China [46]. However, the external input hypothesis was at least argued by the following three archaeology facts. First, there is an absence of strong evidence regarding the observation that domestic horses did not exist before 3300BP. Horse bones, although scarce and broken, were discovered at Pleistocene and Neolithic sites across China [20]. It is difficult to label these bones as domestic or wild based on morphology criteria since horses have not changed much physically as a result of domestication [47]. Horses were not offered as sacrifices until Late Shang Era [20]. That’s one of the reasons that horse remains were a sparse contrast to other livestock in sacrificial places at archaeological sites before Late Shang Dynasty. And husbandry system for horse established over 3000 years ago was strictly controlled over by the royal regime [48]. As a result, horse remains cannot be found in typical tombs. Second, horse riding probably came first preceding driving [49]. It has to be noted that, evaluating the time of horse domestication based on the carriages excavated at the archaeological pits is not an appropriate method. Third, horse archaeological remains have been unearthed in a few parts of southern China [50]. Only one research well studied the ancient DNA of the remains unearthed in northern China [51]. Because of the lack of systematic and reliable evaluation method for distinguishing the domestic and the wild horse by bone fragments, Chinese archaeologists concentrate more on horse gear and carriage [52]. Comprehensive ancient DNA research of horse remains will help us to get a full picture of the origin of the East Asian domestic horse.

Genetics research gives us the possibility to test the external input hypothesis, puzzling the archaeologists and historians. If the genetic components of domestic horses in East Asia are absolutely a subset descended from the outside populations, the external input hypothesis is demonstrable. Europe is an important region for horse domestication because most haplotypes can be traced back to Europe [5]. However, just seven of 23 native horse breeds in China were analyzed in their study. The Iberian Peninsula was provided as refugia for wild horses in the Holocene [6]. The study agrees with the view that some horse haplogroups originated in Europe and then were introduced eastward. Haplogroups I, L and N are prevalent in Europe (Fig. 4, Additional file 8: Table S8, Additional file 15: Figure S4). The association of the western part of Eurasia (Central and West Asia and Europe) mtDNA types was extremely significant in haplogroup I (*P* = 3.96E-12), haplogroup L (*P* = 3.8E-17) and haplogroup N (*P* = 6.35E-10) under the Pearson χ^2^ test (Table 2). The F% (the ratio of the number of unique haplotype to the number of haplotypes) value of the three haplogroups is lower in East Asia (42.9–57.14%) than that in Europe (52.94–72.7%) (Additional file 9: Table S9). And most ancient haplotypes for haplogroups I and L are traced back to the western part of Eurasia (Fig. 3). It is worth to note that three of the five Europe predominant haplotypes (sample size >40) are assigned to haplogroup L, none of Europe predominant haplotypes belongs to haplogroup I. Thus, researchers believe the in-flow of haplogroup L from Europe into East Asia, which is emphasized by nucleotide diversity following the West-to-East gradient (Additional file 9: Table S9).

### Testing the local origin hypothesis

The possibility of the local origin of the horse in East Asia may not be excluded, following history and archaeology records. As indicated in paleontology and history research, preconditions for horse domestication, such as wild horses and vegetation resource, were sufficient in East Asia during the Pleistocene [24, 25, 45, 53]. That is why Eastern steppes and Iberian were refugia for horses in Holocene [6]. The dog and pig domestication were proposed to be under the impact of rice planting culture in East Asia [26, 28]. The study has reason to believe that indigenous horse domestication is likely to occur in East Asia since flourishing grassland culture has been evidenced in this region. Three Neolithic sites (Xinglongwa: 8000BP; Zhaobaogou: 7000BP; Hongshan: 6000-5500BP) representing early grassland culture, were discovered in Inner Mongolia, China [54, 55]. Besides, rock arts representing the herding and riding of horse have been found in the northern part of East Asia at dates before 3600BP [20].

The view of the local origin of the horse in East Asia is reinforced by molecular evidence. Given the indigenous domestication of the horse in East Asia, East Asian-dominating haplogroups and their specific genetic component should be detected. Central and West Asia, Europe and East Asia are three putative horse domestication regions [7]. As shown in Table S8, samples quantity variance are not significant differences between the eastern part of Eurasia (East Asia, 1641 samples) and the western part of Eurasia (Europe and Central and West Asia, 1595 samples). However, haplogroups OP, Q, and R are frequently presented in East Asia (Additional file 8: Table S8, Fig. 4, Additional file 15: Figure S4). Also, a lot of unique haplotypes of East Asia were observed in haplogroups OP, Q, and R (Fig. 3 and Additional file 15: Figure S4). The ratio of the number of unique haplotype to the number of haplotypes (F%) of haplogroups OP, Q, and R are at least about two times more in the eastern part of Eurasia (52.94–72.7%) than that in the western part of Eurasia (18.75–37.5%) (Additional file 9: Table S9). It can be seen that ancient haplotypes for haplogroups Q and R are traced back to East Asia (Fig. 3).

To add credibility and supporting evidence, the ancient domestic horse remains should be considered. One previous ancient DNA research about Chinese horse revealed that clade F (correspond to haplogroups OP and Q now) was presented in samples older than 4000 years [51]. When the remains were assigned according to specific mutation motifs of mtDNA in this study, it was found that ancient domestic horse remains of haplogroup Q are more frequently discovered in East Asia than in other regions. Although haplogroup OP frequently distributes in East Asia (Additional file 8: Table S8 and Additional file 9: S9), East Asia is more likely to be a refugium but not an origin region for haplogroup OP because of its shared ancestral haplotypes that were found both in West Asia and East Asia (Fig. 3). The study did not deny that horse domestication originated in the western part of the Eurasian steppe [7], but haplogroups Q and R originated in East Asia.

To find out the most likely origin region for haplogroup Q, researchers carefully analyzed the 18 unique haplotypes of this haplogroup. In contrast to the sample distribution of haplogroup Q in the SEA and the NEA (Additional file 8: Table S8), unique haplotypes belonging to the SEA have numerical superiority. Nine of the 18 unique haplotypes came from the SEA, while six of the 18 came from the NEA, the rest three were from both the SEA and the NEA. Hence the SEA was presumed as the origin region of haplogroup Q. The nucleotide diversity value of haplogroup R follows the East-to-West gradient (Additional file 9: Table S9). And the F% of haplogroup R is almost about three times in the eastern part of Eurasia as many as that in the western part of Eurasia. The study also analyzed the eight unique haplotypes of haplogroup R to find out the most likely origin region for this haplogroup. Five of the eight unique haplotypes came from the SEA, while two of the eight came from the NEA, the remaining one was from both the SEA and the NEA. Thus researchers infer that the origin of haplogroup R was in the SEA. However, the association of East Asian mtDNA types belonging to haplogroup R was not significant using the Pearson χ^2^ test (*P* = 0.0969) (Table 2). One reasonable explanation is that their gene flow narrowed the genetic population difference between the SEA and the NEA. What is more interesting is one of the 17 haplotypes of early domestic horses that were extinct during the last 5500 years [5] still survives in horse population of southeast China (locate in Dali, Yunnan province).

Moreover, it is essential to note that a few East Asian unique haplotypes show several mutation steps from ancestral haplotypes (Fig. 3). These mutation steps were more likely from local wild horses’ introgression rather than domestication and subsequent evolution. Thus, hybridization probably has contributed new genetic composition to maternal lineages in East Asian domestic horses, but not predominantly.

### Genetic and phylogeographic structure of the domestic horse in East Asia

The genetic and phylogeographic structure of domestic horse in the NEA is the difference from that in the SEA, which is supported by the PCA, haplogroup distribution frequency, network, contour maps of haplogroup frequencies, gene flow analyses, Pearson χ2 test, and AMOVA. From the region-based principal component analysis, most of the haplogroups occur in the NEA and the SEA shows different distributions. It reveals the genetic difference between northern East Asia and southern East Asia (Fig. 2 and Additional file 16: Figure S5). The difference between the NEA and the SEA was proven by further AMOVA analysis. The variation between the NEA and the SEA is significant (*P* = 0.036), although only explaining a low level of genetic variation (1.35%), suggesting the NEA horse populations demonstrates some geographic clustering to the exclusion of the SEA horse populations. Besides, the haplogroup frequency distribution (Additional file 10: Table S10), the minimum spanning network (Additional file 14: Figure S3) and the contour map (Fig. 4), they all show the genetic and phylogeographic difference between the NEA and the SEA. The frequency distribution of haplogroups I, N, OP and Q in the NEA are higher than that in the SEA. However, the frequency distribution of haplogroups H, L, M and QR in the SEA are higher than that in the NEA, and especially haplogroup N is prevalent in the NEA. According to the Pearson χ2 test, the independence for haplogroups EFG, H, I, M, N, OP and R sequences in the NEA and the SEA populations were highly significant (Table 2). Another interesting result is that one of the 12 universally occurring haplotypes (haplotye14, sample size = 88) cannot be found in the SEA horse populations, adding support to the genetic differentiation between the NEA and the SEA.

Based on the gene flow analysis (Additional file 7: Table S7), compared to the SEA populations, relatively large gene flow values were detected between the NEA populations and most non-EA horse populations. In addition, the ratio of the number of unique haplotype to the amount of the haplotype is 44% in the NEA (Additional file 11: Table S11), the proportion of individuals having 12 universally occurring haplotypes (UT) is 39.3% (Additional file 11: Table S11). However, the NEA populations are relatively far from other non-EA populations in the PCA plots. Although different kinds of human action influenced horse gene flow for thousands of years in Eurasia [56, 57]. Researchers speculate that local origin and distinct genetic elements from wild horses involved in the domestication of the NEA horse populations. A previous study indicated multiple ancient DNA of remains unearthed in the NEA region [51], which support our above view to some extent.

In contrast to the NEA, there is relatively week gene flow between the SEA populations and most non-EA horse populations, the ratio of the number of unique haplotype to the number of haplotypes is 55% in the SEA (Additional file 11: Table S11), the proportion of individuals having 12 universally occurring haplotypes (UT) value is 45.1% (Additional file 11: Table S11), however the SEA populations are relatively close to other non-EA populations in the PCA plots. Researchers consider that local origin and a lot of distinct genetic components from wild horses also involved in the domestication of the SEA horse populations. And then genetic barriers occurred between the SEA populations and most non-EA horse populations due to the mountain environment. Another interesting observation is that a sample from Dali (the SEA, Yunnan, China) is one of the 17 extinct haplotypes mentioned by [5], which confirmed gene flow between the SEA and non-EA horse populations that took place in the early stage during domestication.

## Conclusion

Based on high-resolution phylogeny and fine-scale sampling, the investigation of horse complete mitochondrial genome and HVR1 sequences reveals a clear domestication scenario of horse in East Asia: (1) Haplogroup L is inputted from Europe into East Asia; (2) East Asia is more likely to be a refugium but not an origin region for haplogroup OP; (3) Haplogroups Q and R originated in East Asia; (4) Genetic differences were detected between northern East Asia and southern East Asia; (5) The SEA was presumed as the origin region of haplogroup Q and R.

## Supplementary information

**Additional file 1: Table S1.** GenBank entries for novel sequences in this study.

**Additional file 2: Table S2.** Samples information.

**Additional file 3: Table S3.** The definition of geographic regions.

**Additional file 4: Table S4.** PCR and sequencing protocol. Note: 5 ml 10X reaction buffer, 1.5 mM MgCl_2_, 200 μM dNTPs, 10pM each primer, 1 U Taq DNA polymerase (TaKaRa Biosystems). *Kavar T, Bremb G, Habe F, et al., (2002) History of Lipizzan horse maternal lines as revealed by mtDNA analysis. Genet. Sel. Evol. 34: 635–648.

**Additional file 5: Table S5.** A comparison between the new and the old nomenclatures for horse mtDNA haplogroups.

**Additional file 6: Table S6.** Results of BMCMC (Bayesian Markov Chain Monte Carlo) with RLGC-S (relaxed lognormal clock rate and population size estimated from Bayesian skyline plot) model.

**Additional file 7: Table S7.** Matrix of the gene flows (*N*_m_) (below diagonal) among 14 populations of the domestic horse across the world.

**Additional file 8: Table S8.** Dominant haplotypes distribution of haplogroups in horse populations.

**Additional file 9: Table S9.** Genetic statistics for haplogroups in Europe, Central-West Asia, and East Asia modern horse populations 100,000 resampling.

**Additional file 10: Table S10.** Sample size and haplogroup frequency distribution of domestic horse in East Asia.

**Additional file 11: Table S11.** Genetic diversity for control-region sequences of the modern domestic horse across the world (DOC 44 kb)

**Additional file 12: Figure S1.** The choice criterion for sequencing mitochondrial genome sequence in this study.

**Additional file 13: Figure S2.** Classification tree of complete mtDNAs analyzed in this study. Suffixes A, C, G, and T indicate transversions, d indicates deletion; recurrent mutations are underlined; + indicates insertion. The prefix h indicates heteroplasmy and @ highlights back mutation. Numbers in circles are consistent with Additional file 4: Table S4.

**Additional file 14: Figure S3.** Minimum spanning network of haplogroups in East Asia.

**Additional file 15: Figure S4.** Haplotype frequency in three putative horse domestication regions (Europe, Central and West Asia, East Asia).

**Additional file 16: Figure S5.** Region-based PC analysis of mtDNA haplogroup profiles in East Asia**.** (a) PC map of the 13 world horse populations (EE, NE, WE, SE, CE, CA, WA, NA, EA, SA, AF, NAM, SAM) based on haplogroup frequencies. (b) PC map of the 18 world horse populations based on haplogroup frequencies. EA is divided into subpopulations NC, NWC, NEC, SWC, CC, WC. (b) PC map of the East Asia horse populations based on haplogroup frequencies. For more details, see Table 1.

## Data Availability

All novel sequences have been submitted to GenBank (please see the detail in additional file 1: Table S1).

## Abbreviations

AF: Africa
AMOVA: The analysis of molecular variance
BEAST: Bayesian evolutionary analysis sampling trees
BMCMC: Bayesian Markov Chain Monte Carlo
CA: Central Asia
CC: Central China
CE: Central Europe
CR: Control region
EA: East Asia
EE: East Europe
ESS: The estimated sample size
FE: Far East
HVR1: The first hypervariable region
KYA: Thousand years ago
MG: The Republic of Mongolia
MP: Maximum parsimony
NA: North Asia
NAM: North America
NC: North China
NE: North Europe
NEA: The northern East Asia
NEC: Northeast China
NWC: Northwest China
PCA: The principal component analysis
REC-S: The relaxed exponential clock rate and population size determined from Bayesian skyline plot
RLGC-S: The relaxed lognormal clock rate and population size estimated from Bayesian skyline plot
SA: South Asia
SAM: South America
SC-C: The strict clock rate and constant population size
SC-E: The tight clock rate and exponential population size
SC-EXPA: The strict clock rate and expansion population size
SC-S: The strict clock rate and population size estimated from Bayesian skyline plot
SE: South Europe
SEA: The southern East Asia
SWC: Southwest China
TBR: Tree-bisection-reconnection
TMRCA: The time to a most recent common ancestor
UT: The proportion of individuals having 12 universally occurring haplotypes
WA: West Asia
WC: West China
WE: West Europe
