# Supplementary material for: Local origin or external input: modern horse origin in East Asia
Source: BMC Evol Biol. 2019 Nov 27;19:217. doi: 10.1186/s12862-019-1532-y (PMC6882189; doi:10.1186/s12862-019-1532-y)
Supplement: Supplementary file 1 — Additional file 1: Table S1. GenBank entries for novel sequences in this study. [file 12862_2019_1532_MOESM1_ESM.doc]

**Additional file 1: Table S1** GenBank entries for novel sequences in this study

| **HVR1** | |
| --- | --- |
| HAP1 | EU433687 |
| HAP2 | EU433688 |
| HAP3 | EU433689 |
| HAP4 | EU433690 |
| HAP5 | EU433691 |
| HAP6 | EU433692 |
| HAP7 | EU433693 |
| HAP8 | EU433694 |
| HAP9 | EU433695 |
| HAP10 | EU433696 |
| HAP11 | EU433697 |
| HAP12 | EU433698 |
| HAP13 | EU433699 |
| HAP14 | EU433700 |
| HAP15 | EU433701 |
| HAP16 | EU433702 |
| HAP17 | EU433703 |
| HAP18 | EU433704 |
| HAP19 | EU433705 |
| HAP20 | EU433706 |
| HAP21 | EU433707 |
| HAP22 | EU433708 |
| HAP23 | EU433709 |
| HAP24 | EU433710 |
| HAP25 | EU433711 |
| HAP26 | EU433712 |
| HAP27 | EU433713 |
| HAP28 | EU433714 |
| HAP29 | EU433715 |
| HAP30 | EU433716 |
| HAP31 | EU433717 |
| HAP32 | EU433718 |
| HAP33 | EU433719 |
| HAP34 | EU433720 |
| HAP35 | EU433721 |
| HAP36 | EU433722 |
| HAP37 | EU433723 |
| HAP38 | EU433724 |
| HAP39 | EU433725 |
| HAP40 | EU433726 |
| HAP41 | EU433727 |
| HAP42 | EU433728 |
| HAP43 | EU433729 |
| HAP44 | EU433730 |
| HAP45 | EU433731 |
| HAP46 | EU433732 |
| HAP47 | EU433733 |
| HAP48 | EU433734 |
| HAP49 | EU433735 |
| HAP50 | EU433736 |
| HAP51 | EU433737 |
| HAP52 | EU433738 |
| HAP53 | EU433739 |
| HAP54 | EU433740 |
| HAP55 | EU433741 |
| HAP56 | EU433742 |
| HAP57 | EU433743 |
| HAP58 | EU433744 |
| HAP59 | EU433745 |
| HAP60 | EU433746 |
| HAP61 | EU433747 |
| HAP62 | EU433748 |
| HAP63 | EU433749 |
| HAP64 | EU433750 |
| HAP65 | EU433751 |
| HAP66 | EU433752 |
| HAP67 | EU433753 |
| HAP68 | EU433754 |
| HAP69 | EU433755 |
| HAP70 | EU433756 |
| HAP71 | EU433757 |
| HAP72 | EU433758 |
| HAP73 | EU433759 |
| HAP74 | EU433760 |
| HAP75 | EU433761 |
| HAP76 | EU433762 |
| HAP77 | EU433763 |
| HAP78 | EU433764 |
| HAP79 | EU433765 |
| HAP80 | EU433766 |
| HAP81 | EU433767 |
| HAP82 | EU433768 |
| HAP83 | EU433769 |
| HAP84 | EU433770 |
| HAP85 | EU433771 |
| HAP86 | EU433772 |
| HAP87 | EU433773 |
| HAP88 | EU433774 |
| HAP89 | EU433775 |
| HAP90 | EU433776 |
| HAP91 | EU433777 |
| HAP92 | EU433778 |
| HAP93 | EU433779 |
| HAP94 | EU433780 |
| HAP95 | EU433781 |
| HAP96 | EU433782 |
| HAP97 | EU433783 |
| HAP98 | EU433784 |
| HAP99 | EU433785 |
| HAP100 | EU433786 |
| HAP101 | EU433787 |
| HAP102 | EU433788 |
| HAP103 | EU433789 |
| HAP104 | EU433790 |
| HAP105 | EU433791 |
| HAP106 | EU433792 |
| HAP107 | EU433793 |
| HAP108 | EU433794 |
| HAP109 | EU433795 |
| HAP110 | EU433796 |
| HAP111 | EU433797 |
| HAP112 | EU433798 |
| HAP113 | EU433799 |
| HAP114 | EU433800 |
| HAP115 | EU433801 |
| HAP116 | EU433802 |
| HAP117 | EU433803 |
| HAP118 | EU433804 |
| HAP119 | EU433805 |
| HAP120 | EU433806 |
| HAP121 | EU433807 |
| HAP122 | EU433808 |
| HAP123 | EU433809 |
| HAP124 | EU433810 |
| HAP125 | EU433811 |
| HAP126 | EU433812 |
| HAP127 | EU433813 |
| HAP128 | EU433814 |
| HAP129 | EU433815 |
| HAP130 | EU433816 |
| HAP131 | EU433817 |
| HAP132 | EU433818 |
| HAP133 | EU433819 |
| HAP134 | EU433820 |
| HAP135 | EU433821 |
| HAP136 | EU433822 |
| HAP137 | EU433823 |
| HAP138 | EU433824 |
| HAP139 | EU433825 |
| HAP140 | EU433826 |
| HAP141 | EU433827 |
| HAP142 | EU433828 |
| HAP143 | EU433829 |
| HAP144 | EU433830 |
| HAP145 | EU433831 |
| HAP146 | EU433832 |
| HAP147 | EU433833 |
| HAP148 | EU433834 |
| HAP149 | EU433835 |
| HAP150 | EU433836 |
| HAP151 | EU433837 |
| HAP152 | EU433838 |
| HAP153 | EU433839 |
| HAP154 | JQ360633 |
| HAP155 | JQ360634 |
| HAP156 | JQ360635 |
| HAP157 | JQ360636 |
| HAP158 | JQ360637 |
| HAP159 | JQ360638 |
| HAP160 | JQ360639 |
| HAP161 | JQ360640 |
| HAP162 | JQ360641 |
| HAP163 | JQ360642 |
| HAP164 | JQ360643 |
| HAP165 | JQ360644 |
| HAP166 | JQ360645 |
| HAP167 | JQ360646 |
| HAP168 | JQ360647 |
| HAP169 | JQ360648 |
| HAP170 | JQ360649 |
| HAP171 | JQ360650 |
| HAP172 | JQ360651 |
| HAP173 | JQ360652 |
| HAP174 | JQ360653 |
| HAP175 | JQ360654 |
| HAP176 | JQ360655 |
| HAP177 | JQ360656 |
| HAP178 | JQ360657 |
| HAP179 | JQ360658 |
| HAP180 | JQ360659 |
| HAP181 | JQ360660 |
| HAP182 | JQ360661 |
| HAP183 | JQ360662 |
| HAP184 | JQ360663 |
| HAP185 | JQ360664 |
| HAP186 | JQ360665 |
| HAP187 | JQ360666 |
| HAP188 | JQ360667 |
| HAP189 | JQ360668 |
| HAP190 | JQ360669 |
| HAP191 | JQ360670 |
| HAP192 | JQ360671 |
| HAP193 | JQ360672 |
| HAP194 | JQ360673 |
| HAP195 | JQ360674 |
| HAP196 | JQ360675 |
| HAP197 | JQ360676 |
| HAP198 | JQ360677 |
| HAP199 | JQ360678 |
| HAP200 | JQ360679 |
| HAP201 | JQ360680 |
| HAP202 | JQ360681 |
| HAP203 | JQ360682 |
| HAP204 | JQ360683 |
| HAP205 | JQ360684 |
| HAP206 | JQ360685 |
| HAP207 | JQ360686 |
| HAP208 | JQ360687 |
| HAP209 | JQ360688 |
| HAP210 | JQ360689 |
| HAP211 | JQ360690 |
| HAP212 | JQ360691 |
| HAP213 | JQ360692 |
| HAP214 | JQ360693 |
| HAP215 | JQ360694 |
| HAP216 | JQ360695 |
| HAP217 | JQ360696 |
| HAP218 | JQ360697 |
| HAP219 | JQ360698 |
| HAP220 | JQ360699 |
| HAP221 | JQ360700 |
| HAP222 | JQ360701 |
| HAP223 | JQ360702 |
| HAP224 | JQ360703 |
| HAP225 | JQ360704 |
| HAP226 | JQ360705 |
| HAP227 | JQ360706 |
| HAP228 | JQ360707 |
| HAP229 | JQ360708 |
| HAP230 | JQ360709 |
| HAP231 | JQ360710 |
| HAP232 | JQ360711 |
| HAP233 | JQ360712 |
| HAP234 | JQ360713 |
| HAP235 | JQ360714 |
| HAP236 | JQ360715 |
| HAP237 | JQ360716 |
| HAP238 | JQ360717 |
| HAP239 | JQ360718 |
| HAP240 | JQ360719 |
| HAP241 | JQ360720 |
| HAP242 | JQ360721 |
| HAP243 | JQ360722 |
| HAP244 | JQ360723 |
| HAP245 | JQ360724 |
| HAP246 | JQ360725 |
| HAP247 | JQ360726 |
| HAP248 | JQ360727 |
| HAP249 | JQ360728 |
| HAP250 | JQ360729 |
| HAP251 | JQ360730 |
| HAP252 | JQ360731 |
| HAP253 | JQ360732 |
| HAP254 | JQ360733 |
| HAP255 | JQ360734 |
| HAP256 | JQ360735 |
| HAP257 | JQ360736 |
| HAP258 | JQ360737 |
| HAP259 | JQ360738 |
| HAP260 | JQ360739 |
| HAP261 | JQ360740 |
| HAP262 | JQ360741 |
| HAP263 | JQ360742 |
| HAP264 | JQ360743 |
| HAP265 | JQ360744 |
| HAP266 | JQ360745 |
| HAP267 | JQ360746 |
| HAP268 | JQ360747 |
| HAP269 | JQ360748 |
| HAP270 | JQ360749 |
| HAP271 | JQ360750 |
| HAP272 | JQ360751 |
| HAP273 | JQ360752 |
| HAP274 | JQ360753 |
| HAP275 | JQ360754 |
| HAP276 | JQ360755 |
| HAP277 | JQ360756 |
| HAP278 | JQ360757 |
| HAP279 | JQ360758 |
| HAP280 | JQ360759 |
| HAP281 | JQ360760 |
| HAP282 | JQ360761 |
| HAP283 | JQ360762 |
| HAP284 | JQ360763 |
| HAP285 | JQ360764 |
| HAP286 | JQ360765 |
| HAP287 | JQ360766 |
| HAP288 | JQ360767 |
| HAP289 | JQ360768 |
| HAP290 | JQ360769 |
| HAP291 | JQ360770 |
| HAP292 | JQ360771 |
| HAP293 | JQ360772 |
| HAP294 | JQ360773 |
| HAP295 | JQ360774 |
| HAP296 | JQ360775 |
| HAP297 | JQ360776 |
| HAP298 | JQ360777 |
| HAP299 | JQ360778 |
| HAP300 | JQ360779 |
| HAP301 | JQ360780 |
| HAP302 | JQ360781 |
| HAP303 | JQ360782 |
| HAP304 | JQ360783 |
| HAP305 | JQ360784 |
| HAP306 | JQ360785 |
| HAP307 | JQ360786 |
| HAP308 | JQ360787 |
| HAP309 | JQ360788 |
| HAP310 | JQ360789 |
| HAP311 | JQ360790 |
| HAP312 | JQ360791 |
| HAP313 | JQ360792 |
| HAP314 | JQ360793 |
| HAP315 | JQ360794 |
| HAP316 | JQ360795 |
| HAP317 | JQ360796 |
| HAP318 | JQ360797 |
| HAP319 | JQ360798 |
| HAP320 | JQ360799 |
| HAP321 | JQ360800 |
| HAP322 | JQ360801 |
| HAP323 | JQ360802 |
| HAP324 | JQ360803 |
| HAP325 | JQ360804 |
| HAP326 | JQ360805 |
| HAP327 | JQ360806 |
| HAP328 | JQ360807 |
| HAP329 | JQ360808 |
| HAP330 | JQ360809 |
| HAP331 | JQ360810 |
| HAP332 | JQ360811 |
| HAP333 | JQ360812 |
| HAP334 | JQ360813 |
| HAP335 | JQ360814 |
| HAP336 | JQ360815 |
| HAP337 | JQ360816 |
| HAP338 | JQ360817 |
| HAP339 | JQ360818 |
| HAP340 | JQ360819 |
| HAP341 | JQ360820 |
| HAP342 | JQ360821 |
| HAP343 | JQ360822 |
| HAP344 | JQ360823 |

| **ATP6** | |
| --- | --- |
| hap1 | FJ765155 |
| hap2 | FJ765156 |
| hap3 | FJ765157 |
| hap4 | FJ765158 |
| hap5 | FJ765159 |
| hap6 | FJ765160 |
| hap7 | FJ765161 |
| hap8 | FJ765162 |
| hap9 | FJ765163 |
| hap10 | FJ765164 |
| hap11 | FJ765165 |
| hap12 | FJ765166 |
| hap13 | FJ765167 |
| hap14 | FJ765168 |
| hap15 | FJ765169 |
| hap16 | FJ765170 |
| hap17 | FJ765171 |
| hap18 | FJ765172 |
| hap19 | FJ765173 |
| hap20 | FJ765174 |
| hap21 | FJ765175 |
| hap22 | FJ765176 |
| hap23 | FJ765177 |
| hap24 | FJ765178 |
| hap25 | FJ765179 |
| hap26 | FJ765180 |
| hap27 | FJ765181 |
| hap28 | FJ765182 |
| hap29 | FJ765183 |
| hap30 | FJ765184 |
| hap31 | FJ765185 |
| hap32 | FJ765186 |
| hap33 | FJ765187 |
| hap34 | FJ765188 |
| hap35 | FJ765189 |
| hap36 | FJ765190 |
| hap37 | FJ765191 |
| hap38 | FJ765192 |
| hap39 | FJ765193 |
| hap40 | FJ765194 |
| hap41 | FJ765195 |
| hap42 | FJ765196 |
| hap43 | FJ765197 |
| hap44 | FJ765198 |
| hap45 | FJ765199 |
| hap46 | FJ765200 |
| hap47 | FJ765201 |
| hap48 | FJ765202 |
| hap49 | FJ765203 |
| hap50 | FJ765204 |
| hap51 | FJ765205 |
| hap52 | FJ765206 |
| hap53 | FJ765207 |
| hap54 | FJ765208 |

| **ATP8** | |
| --- | --- |
| hap1 | FJ643628 |
| hap2 | FJ643629 |
| hap3 | FJ643630 |
| hap4 | FJ643631 |
| hap5 | FJ643632 |
| hap6 | FJ643633 |
| hap7 | FJ643634 |
| hap8 | FJ643635 |
| hap9 | FJ643636 |
| hap10 | FJ643637 |
| hap11 | FJ643638 |
| hap12 | FJ643639 |
| hap1 | FJ643640 |
| hap2 | FJ643641 |
| hap3 | FJ643642 |
| hap4 | FJ643643 |
| hap5 | FJ643644 |
| hap6 | FJ643645 |
| hap7 | FJ643646 |
| hap8 | FJ643647 |
| hap9 | FJ643648 |
| hap10 | FJ643649 |
| hap11 | FJ643650 |
| hap12 | FJ643651 |
| hap13 | FJ643652 |
| hap14 | FJ643653 |
| hap15 | FJ643654 |
| hap16 | FJ643655 |
| hap17 | FJ643656 |
| hap18 | FJ643657 |
| hap19 | FJ643658 |
| hap20 | FJ643659 |
| hap21 | FJ643660 |
| hap22 | FJ643661 |
| hap23 | FJ643662 |
| hap24 | FJ643663 |
| hap25 | FJ643664 |
| hap26 | FJ643665 |
| hap27 | FJ643666 |
| hap28 | FJ643667 |
| hap29 | FJ643668 |
| hap30 | FJ643669 |
| hap31 | FJ643670 |
| hap32 | FJ643671 |
| hap33 | FJ643672 |
| hap34 | FJ643673 |
| hap35 | FJ643674 |
| hap36 | FJ643675 |

| **CYTB** | |
| --- | --- |
| Hap_1 | EU433643 |
| Hap_2 | EU433644 |
| Hap_3 | EU433645 |
| Hap_4 | EU433646 |
| Hap_5 | EU433647 |
| Hap_6 | EU433648 |
| Hap_7 | EU433649 |
| Hap_8 | EU433650 |
| Hap_9 | EU433651 |
| Hap_10 | EU433652 |
| Hap_11 | EU433653 |
| Hap_12 | EU433654 |
| Hap_13 | EU433655 |
| Hap_14 | EU433656 |
| Hap_15 | EU433657 |
| Hap_16 | EU433658 |
| Hap_17 | EU433659 |
| Hap_18 | EU433660 |
| Hap_19 | EU433661 |
| Hap_20 | EU433662 |
| Hap_21 | EU433663 |
| Hap_22 | EU433664 |
| Hap_23 | EU433665 |
| Hap_24 | EU433666 |
| Hap_25 | EU433667 |
| Hap_26 | EU433668 |
| Hap_27 | EU433669 |
| Hap_28 | EU433670 |
| Hap_29 | EU433671 |
| Hap_30 | EU433672 |
| Hap_31 | EU433673 |
| Hap_32 | EU433674 |
| Hap_33 | EU433675 |
| Hap_34 | EU433676 |
| Hap_35 | EU433677 |
| Hap_36 | EU433678 |
| Hap_37 | EU433679 |
| Hap_38 | EU433680 |
| Hap_39 | EU433681 |
| Hap_40 | EU433682 |
| Hap_41 | EU433683 |
| Hap_42 | EU433684 |
| Hap_43 | EU433685 |
| Hap_44 | EU433686 |
| Hap_45 | FJ765096 |
| Hap_46 | FJ765097 |
| Hap_47 | FJ765098 |
| Hap_48 | FJ765099 |
| Hap_49 | FJ765100 |
| Hap_50 | FJ765101 |
| Hap_51 | FJ765102 |
| Hap_52 | FJ765103 |
| Hap_53 | FJ765104 |
| Hap_54 | FJ765105 |
| Hap_55 | FJ765106 |
| Hap_56 | FJ765107 |
| Hap_57 | FJ765108 |
| Hap_58 | FJ765109 |
| Hap_59 | FJ765110 |
| Hap_60 | FJ765111 |
| Hap_61 | FJ765112 |
| Hap_62 | FJ765113 |
| Hap_63 | FJ765114 |
| Hap_64 | FJ765115 |
| Hap_65 | FJ765116 |
| Hap_66 | FJ765117 |
| Hap_67 | FJ765118 |
| Hap_68 | FJ765119 |
| Hap_69 | FJ765120 |
| Hap_70 | FJ765121 |
| Hap_71 | FJ765122 |
| Hap_72 | FJ765123 |
| Hap_73 | FJ765124 |
| Hap_74 | FJ765125 |
| Hap_75 | FJ765126 |
| Hap_76 | FJ765127 |
| Hap_77 | FJ765128 |
| Hap_78 | FJ765129 |
| Hap_79 | FJ765130 |
| Hap_80 | FJ765131 |
| Hap_81 | FJ765132 |
| Hap_82 | FJ765133 |
| Hap_83 | FJ765134 |
| Hap_84 | FJ765135 |
| Hap_85 | FJ765136 |
| Hap_86 | FJ765137 |
| Hap_87 | FJ765138 |
| Hap_88 | FJ765139 |
| Hap_89 | FJ765140 |
| Hap_90 | FJ765141 |
| Hap_91 | FJ765142 |
| Hap_92 | FJ765143 |
| Hap_93 | FJ765144 |
| Hap_94 | FJ765145 |
| Hap_95 | FJ765146 |
| Hap_96 | FJ765147 |
| Hap_97 | FJ765148 |
| Hap_98 | FJ765149 |
| Hap_99 | FJ765150 |
| Hap_100 | FJ765151 |
| Hap_101 | FJ765152 |
| Hap_102 | FJ765153 |
| Hap_103 | FJ765154 |

| **Mitochondrial genomes** | |
| --- | --- |
| TN8947 | FJ718996 |
| TN9178 | FJ718997 |
| TN9106 | FJ718998 |
| TNTM14 | GU734774 |
| TN8804 | GU734776 |
| TN8809 | GU734777 |
| TN8815 | GU734778 |
| TN8859 | GU734779 |
| TN9411 | GU734780 |
| TN8948 | GU734781 |
| TN8951 | GU734782 |
| TN8968 | GU734783 |
| TN8987 | GU734784 |
| AAPP62 | JQ340091 |
| AAPP84 | JQ340092 |
| AAPP95 | JQ340093 |
| AAPP108 | JQ340094 |
| AAPP109 | JQ340095 |
| AAPP124 | JQ340096 |
| AAPP134 | JQ340097 |
| AAPP159 | JQ340098 |
| AAPP185 | JQ340099 |
| AAPP189 | JQ340100 |
| TN9252 | JQ340101 |
| TN9340 | JQ340102 |
| TN20 | JQ340103 |
| TN8806 | JQ340104 |
| TN8819 | JQ340105 |
| TN8824 | JQ340106 |
| TN8830 | JQ340107 |
| TN8834 | JQ340108 |
| TN8907 | JQ340109 |
| TN8970 | JQ340110 |
| TN8983 | JQ340111 |
| TN8988 | JQ340112 |
| TN8990 | JQ340113 |
| TN8998 | JQ340114 |
| TN9002 | JQ340115 |
| TN9009 | JQ340116 |
| TN9010 | JQ340117 |
| TN9011 | JQ340118 |
| TN9014 | JQ340119 |
| TN9018 | JQ340120 |
| TN9034 | JQ340121 |
| TN9092 | JQ340122 |
| TN9209 | JQ340123 |
| TN9288 | JQ340124 |
| TN9291 | JQ340125 |
| TN9294 | JQ340126 |
| TN9308 | JQ340127 |
| TN9313 | JQ340128 |
| TN9315 | JQ340129 |
| TN9318 | JQ340130 |
| TN9322 | JQ340131 |
| TN9323 | JQ340132 |
| TN9328 | JQ340133 |
| TN9330 | JQ340134 |
| TN9334 | JQ340135 |
| TN9343 | JQ340136 |
| TN9377 | JQ340137 |
| TN9381 | JQ340138 |
| TN9398 | JQ340139 |
| TN9400 | JQ340140 |
| TN9412 | JQ340141 |
| TN9432 | JQ340142 |
| TN9480 | JQ340143 |
| TN9488 | JQ340144 |
| TN9505 | JQ340145 |
| TNDM9 | JQ340146 |
| TNDM10 | JQ340147 |
| TNDM12 | JQ340148 |
| TNDM17 | JQ340149 |
| TNGX1 | JQ340150 |
| TNIn14 | JQ340151 |
| TNIn20 | JQ340152 |
| TNJA2 | JQ340153 |
| TNJA4 | JQ340154 |
| TNJA8 | JQ340155 |
| TNJA9 | JQ340156 |
| TNJA13 | JQ340157 |
| TNQM1 | JQ340158 |
| TNQM8 | JQ340159 |
| TNQM18 | JQ340160 |
| TNSL1 | JQ340161 |
| TNSL3 | JQ340162 |
| TNTM37 | JQ340163 |
| TNTM43 | JQ340164 |
| TNWM12 | JQ340165 |
| TNWM20 | JQ340166 |
| TNXJ4 | JQ340167 |
| AAPP30 | JQ340168 |
| AAPP32 | JQ340169 |
| AAPP166 | JQ340170 |
