# Supplementary material for: Local origin or external input: modern horse origin in East Asia
Source: BMC Evol Biol. 2019 Nov 27;19:217. doi: 10.1186/s12862-019-1532-y (PMC6882189; doi:10.1186/s12862-019-1532-y)
Supplement: Supplementary file 3 — Additional file 3: Table S3. The definition of geographic regions. [file 12862_2019_1532_MOESM3_ESM.doc]

**Additional file 3: Table S3** The definition of geographic regions

**
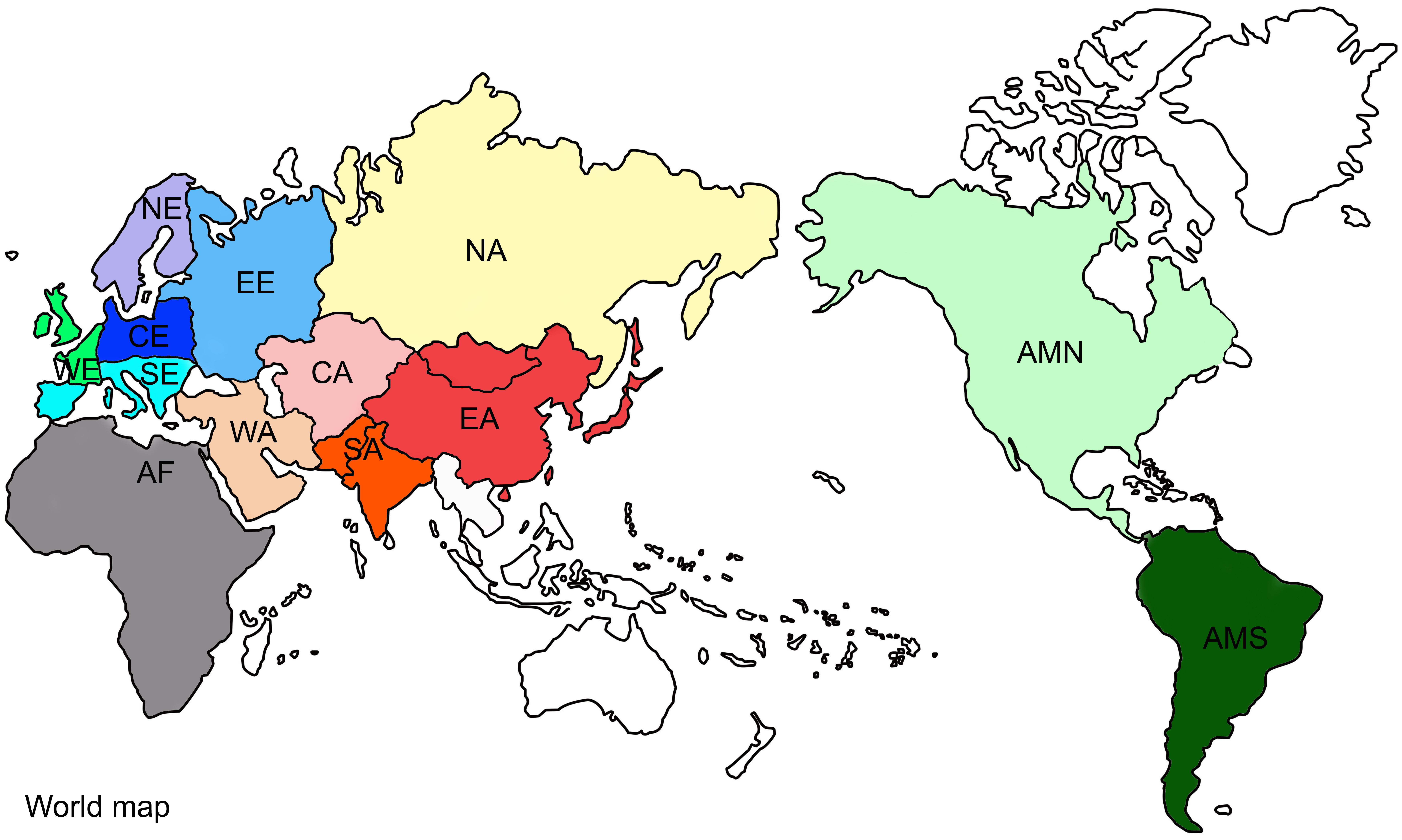
**

**AF** - Africa; **AF** includes Morocco and Egypt.

**NAM** - North America; **NAM** includes United States and Canada.

**SAM** - South America; **SAM** includes Brazil, Chile and Argentina.

**CA -** Central Asia; **CA** includes Kazakhstan, Uzbekistan, Tajikistan, Turkmenistan and Kyrgyzstan.

**EA -** East Asia; **EA** includes China, North Korea, South Korea, Japan and Mongolia.

**NA -** North Asia; **NA** is the Asian part of Russia. The east sides of the Ural River and the Ural Mountains.

**SA** - South Asia; **SA** includes Pakistan, India, Maldives, Sri Lanka, Nepal, Bhutan and Bangladesh.

**WA** - West Asia; **WA** includes Afghanistan, Armenia, Azerbaijan, Bahrain, Cyprus, Georgia, Iran, Iraq, Israel, Jordan, Kuwait, Lebanon, Palestinian Territories, Oman, Qatar, Saudi Arabia, Syria, Turkey, United Arab Emirates and Yemen.

**CE** - Central Europe; **CE** includes Poland, Czech, Slovakia, Hungary, Germany, Austria, Switzerland and Liechtenstein.

**EE** - East Europe; **EE** includes Estonia, Latvia, Belorussia, Ukraine, Moldova and the west sides of the Russian Federation (the west sides of the Ural River and the Ural Mountains).

**NE** - North Europe; **NE** includes Scandinavian Peninsula, Iceland, Denmark, Norway, Sweden, Finland and Lithuania.

**SE** - South Europe; **SE** includes Yugoslavia, Croatia, Slovenia, Bosnian and Herzegovina Hessen, Macedonia, Romania, Bulgaria, Albania, Greece, Italy, Vatican, San Marino, Malta, Spain, Portugal and Andorra.

**WE** - West Europe; **WE** includes Britain, Ireland, Holland, Belgium, Luxembourg, France and Monaco.

**FE** - Far East; **FE** includes Korea, South Korea and Japan.

**MG** –MG is the Republic of Mongolia.

**Note**: We took the outline map of the national basic geographic information center ([http://www.ngcc.cn](http://www.ngcc.cn/)) as the base map, and then drew it ourselves according to the analysis requirements.


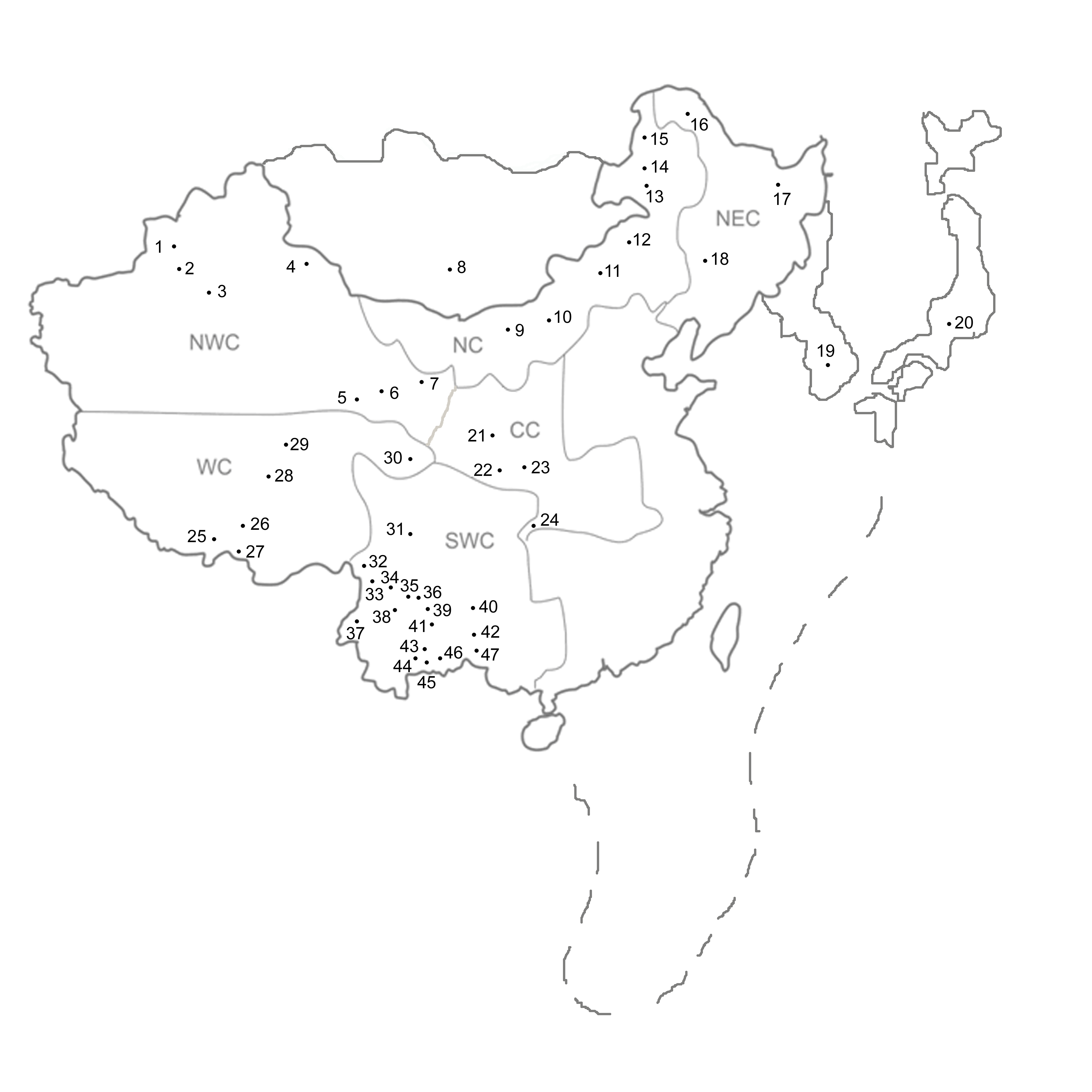


**East Asia domestic horse**

**Abbreviation used for regions: CC** - Central China; **CC** includes Shaanxi, Hubei, Henan and the south part of Gansu. **NC** - North China; **NC** includes Inner Mongolia. **NEC** - Northeast China; **NEC** includes Jilin, Liaoning and Heilongjiang. **NWC** - Northwest China; **NWC** includes Xingjiang, the Northwest part of Qinghai (Dulan, Qilian, Wulan, Delingha, Chaidamu. Datong) and the north part of Gansu. **SWC** - Southwest China; **SWC** includes Sichuan, Yunan, Guangxi, Guizhou and Chongqing. **WC** - West China; **WC** includes Tibet and the west part of Qinghai (Yushu, Henan Hequ and Tongde).

**Abbreviation used for populations: CC** including **(HBL:** Lichuan, **SXGZ:** Guanzhong, **SXNQ:** Ningqiang pony, **GS:** Guanshan**)** populations; **WC** including (**HQ:** Hequ, **JZI:** Jiangzi, **LKZ:** Langkazi, **NIM:** Nimu, **TB:** Tibetan, **YS:**Yushu**)** populations; **NC** including **(BR:** Baerhu, **IMG:** Inner Mongolia, **SH:** Sanhe, **SS:** Sengseng, **WSE:** Wushen, **WZMQ:** Wuzhumuqin, **XNH:** Xinihe**)** populations; **NEC** including **(ELC:** Elunchun, **HH:** Heihe, **JL:** Jilin**)** populations; **NWC** including **(BLK:** Balikun, **CDM:** Chaidamu, **DT:** Datong, **DLH:** Delinha, **KZK:** Kazahk, **YQ:** Yanqi, **YL:** Yili**)** populations; **SWC** including **(ZD:** Zhongdian, **YNT:** Yunnan Tibetan, **YNP:** Yunnan pony, **WM:** Wumeng, **WSA:** Wenshan pony, **TC:** Tenchong, **SCT:** Sichuan Tibetan, **MLP:** Malipo, **MAG:** Maguan pony, **LP:** Luoping, **LJ:** Lijiang, **LH:** Luhe, **JC:** Jiangchang, **GZ:** Guizhou, **DB:** Debao pony, **DAL:** Dali, **BIS:** Baise**)** populations; **FE** including **(JAN:** Japan native, **KJU:** Korea Jeju or Cheju**)** populations; **MG:** Mongolia populations.

**Note**: We took the outline map of the national basic geographic information center ([http://www.ngcc.cn](http://www.ngcc.cn/)) as the base map, and then drew it ourselves according to the analysis requirements.
