# Supplementary material for: Local origin or external input: modern horse origin in East Asia
Source: BMC Evol Biol. 2019 Nov 27;19:217. doi: 10.1186/s12862-019-1532-y (PMC6882189; doi:10.1186/s12862-019-1532-y)
Supplement: Supplementary file 4 — Additional file 4: Table S4. PCR and sequencing protocol. Note: 5 ml 10X reaction buffer, 1.5 mM MgCl2, 200 μM dNTPs, 10pM each primer, 1 U Taq DNA polymerase (TaKaRa Biosystems). *Kavar T, Bremb G, Habe F, et al., (2002) History of Lipizzan horse maternal lines as revealed by mtDNA analysis. Genet. Sel. Evol. 34: 635–648. [file 12862_2019_1532_MOESM4_ESM.doc]

**Additional file 4: Table S4** PCR and sequencing protocol

| Name | 5’ to 3’ | PCR mixture | PCR program | Note |
| --- | --- | --- | --- | --- |
| H1F | GCACTGAAAATGCCTAGATGAGTATTCTT | LA polymerase(5U/μl): 0.5μl  LA buffer(+Mg^2+^, 10X): 5μl  dNTP Mix(2.5mM): 4μl  Primer F(10ρmol/μl): 1μl  Primer R(10ρmol/μl): 1μl  DNA(100ng/μl): 1μl  ddH_2_O: 37.5μl | Step1: 94°C 1min  Step2(30cycles): 98°C 10sec, 65°C 16min,  Step3: 72°C 10min | 1)Blood and tissue samples  2)PCR primers for mitochondrial genomes |
| H1R | TGAGGTGGAATAGGTTAGTCGTATGTAGA |
| H2F | TCTACATACGACTAACCTATTCCACCTCAC |
| H2R | GAAGTTAATCATAGTTTAATGAGTCGAAATCA |
| H3F | ACTCATCGCATTCTGACTACCACAACT |
| H3R | TCCTACGTGAATGAAGAGGCAGATAAA |
| H4F | AAATCAAATTATATTTCCTCTCATTC |
| H4R | GAGTTTTAGGCTGTTGCTAGTAGTAC |
|  | | | | |
| H1QF | ACATGAATCGGCGGACAG | r polymerase(5U/μl): 0.5μl  r buffer(+Mg^2+^, 10X): 5.6μl  dNTP Mix(2.5mM): 2.4μl  Primer F(10ρmol/μl): 1μl  Primer R(10ρmol/μl): 1μl  DNA(100ng/μl): 1μl  ddH_2_O: 38.5μl | Step1: 95°C 3min  Step2(38cycles): 95°C 30sec, 59°C 45sec, 72°C 2min  Step3: 72°C 10min | 1)Hair root and buccal swap samples  2)PCR primers for mitochondrial genomes |
| H1QR | TTAGCAAGGGATGGTGGG |
| H2QF | ATAAAGCAAGGCACTGAA |
| H2QR | ATAGGTAGCTCGTCTGGT |
| H3QF | GCTTACCCCTTTTACCTT |
| H3QR | ATAGAAACCGACCTGGAT |
| H4QF | ACAACCTCCGAGTGATTT |
| H4QR | GGATGATGCTGCTATTTT |
| H5QF | TCTAACCACTACACTAATCT |
| H5QR | ATAAGTATCGTGGAGAAC |
| H6QF | TGCACGGAGGAAATATCAA |
| H6QR | ATTCTCAAGGGGCAGAGTG |
| H7QF | TTTACTACGGTCAATGCTCA |
| H7QR | CCTTCTATTAGGCTATGGTG |
| H8QF | CCACCCACAGGTATCCAC |
| H8QR | AGATCGGTTTGTCGCAAG |
| H9QF | GTTCCATCTGCTTGCGACAA |
| H9QR | TGCCTACTCCCTCCCATCC |
| H10QF | CAACCTTACCAACCTCCCG |
| H10QR | GGTGTTCCACTGGCTGTCC |
| H1B1F | ACATGAATCGGCGGACAG |
| H1B1R | AGGGCTAAGCATAGTGGG |
| H1B2F | TGGTAGCGGGACATAGGA |
| H1B2R | TTAGCAAGGGATGGTGGG |
| H2B1F | ATAAAGCAAGGCACTGAA |
| H2B1R | CTAGGTGTAAGCTGGATG |
| H2B2F | AGGAGGATTTAGCAGTAA |
| H2B2R | TTGCGACATAGATGAGTT |
| H3B1F | AGGGAACGATGAAAGATG |
| H3B1R | CTTTGCACGGTTAGGATA |
| H3B2F | ATCAGAACGAATACTCAC |
| H3B2R | AACGAACCCTTAATAGCG |
| H4B1F | AAACAACCTCCGAGTGAT |
| H4B1R | AAATAAGAGGGTTTGAGC |
| H4B2F | CTCAACATTAGCCGAAAC |
| H4B2R | ATGAATCCTGATAGTGGG |
| H5B1F | TACCTCTAACCACTACACT |
| H5B1R | GTAAATCTAAAGACAGGG |
| H5B2F | TAAATACCCTAATCAACTGG |
| H5B2R | AAGTATCGTGGAGAACAA |
| H6B1F | CGCTATCCCTACTGGTGT |
| H6B1R | AAGGATGATGGCTGGTAA |
| H6B2F | GATTTTACCAGCCATCAT |
| H6B2R | GGGGCAGAGTGTTTTGTT |
| H7B1F | TGCGGATCAAACCACAGCT |
| H7B1R | AGTGTTGCCCCTCCGATGA |
| H7B2F | TTCATTGGCTCAACAAACT |
| H7B2R | GGTGGGCTCAGGTGATAGA |
| H8B1F | CTCTATCACCTGAGCCCACCAT |
| H8B1R | CGTTCGCAGGCAGCAAATAC |
| H8B2F | AGCATTCACAGTATCCCTCGTA |
| H8B2R | TTAGATCGGTTTGTCGCAAG |
| H9B1F | GTTCCATCTGCTTGCGACAA |
| H9B1R | AGGGCCATGAGTGCGTTT |
| H9B2F | TATCACCATCACCGCCCTCT |
| H9B2R | TGCCTACTCCCTCCCATCC |
| H10B1F | TAACCGCATCGGGGATATC |
| H10B1R | GGGCTGGTAGGTCAATAAAAGAG |
| H10B2F | AGTCACAATCACAACCCAAGC |
| H10B2R | GACCAGGGTAATGTGCGATAT |
|  | | | | |
| 115L | ACATGCAAGTATCCGCACCC |  |  | 1)Blood and tissue samples  2)Sequencing primers for mitochondrial genomes |
| 592L | AAGGACTTGGCGGTGCTTTA |
| 925L | ACCGCCCGTCACCCTCCTTA |
| 1233L | TAAGGGAACGATGAAAGATGC |
| 1454H | GACAACCAGCTATCACCAG |
| 2165H | CATAGGGTCTTCTCGTCTTA |
| 2436H | CAACATCGAGGTCGTAAA |
| 2684L | ACAGGGCTCGTTAGGGTGGC |
| 2914H | TTGAGGGCATCGGCAATA |
| 3326L | TCATCTCAACATTAGCCGAAAC |
| 3635L | TATGAAAGAACTTCCTACCAC |
| 4086L | CGAACCATAGAAGCCTCCAC |
| 4425L | GTAGGAGGCTGAGGCGGTCT |
| 5132H | TTCGTGGGATGGTAGCC |
| 5715H | TGTTCCGGCACCTGCTTCA |
| 6092H | CGAAGCCTGGTAGGATAAG |
| 6542H | AGAATAGAGGGATCAGTGTA |
| 6482L | CATTTCCATTATGTCCTG |
| 7419L | CCACATCAGACCTAAAACC |
| 7612L | CAGGACTTTACTACGGTCAATG |
| 7623H | TCTGAGCATTGACCGTAG |
| 7939L | AACACTCTGCCCCTTGA |
| 8439L | GGCTAACCGCTAACATT |
| 8990L | GACCACCCACAGGTATCCA |
| 9240H | AGTCCGTGGAACCCTGT |
| 9541H | TGCGATGAGTACGAGTAGCG |
| 9545L | CTCGTACTCATCGCATTCTG |
| 9830L | AGAATGAACTGAGTATGGTA |
| 10107H | TTCGCAGGCAGCAAATAC |
| 10448L | CACCATCTATCTAAGGAAC |
| 10698L | CTCTATCCAAAACCTAAC |
| 10920H | CGTAGTATTCCGTAGCCTC |
| 11502H | GGCCATGAGTGCGTTTT |
| 12073H | GAATTCCATAATAGATCAT |
| 12342L | CCTGATTCCTATTCAACAC |
| 12542H | AGGAAGACGCCTGCTACA |
| 12732H | ATGGTTACGATTATCAGGC |
| 13204L | CACAACCGTCCCAGAAATA |
| 13390H | AGGTTAGCGAGCGGTGGG |
| 13494H | ATTTGGAACTGGGAGATAG |
| 14127L | GGAATCTAACCACGACCAA |
| 14365H | GGATGAGGAGGCAGTTGT |
| 14616L | AACAGTCATCACGAACCTC |
| 14934L | GGAGACCCAGACAACTACAC |
| 15340L | GCACATTACCCTGGTCTTG |
| 15691L | TTTCCAGTCAACACGCATA |
| 16101L | TGGTAGCGGGACATAGGAA |
| 16511L | ACCATAATAACTCAACACACC |
| 16640H | GAAGAAGGGTTGGACAGAT |
|  | | | | |
| H1B1F | ACATGAATCGGCGGACAG |  |  | 1)Hair root and buccal swap samples  2)Sequencing primers for mitochondrial genomes |
| 15340L | GCACATTACCCTGGTCTTG |
| 15691L | TTTCCAGTCAACACGCATA |
| 16101L | TGGTAGCGGGACATAGGAA |
| 16511L | ACCATAATAACTCAACACACC |
| 16640H | GAAGAAGGGTTGGACAGAT |
| 115L | ACATGCAAGTATCCGCACCC |
| H1B2R | TTAGCAAGGGATGGTGGG |
| H2QF | ATAAAGCAAGGCACTGAA |
| 115L | ACATGCAAGTATCCGCACCC |
| 592L | AAGGACTTGGCGGTGCTTTA |
| 925L | ACCGCCCGTCACCCTCCTTA |
| H2QR | ATAGGTAGCTCGTCTGGT |
| H3QF | GCTTACCCCTTTTACCTT |
| 1233L | TAAGGGAACGATGAAAGATGC |
| 1454H | GACAACCAGCTATCACCAG |
| 2165H | CATAGGGTCTTCTCGTCTTA |
| H3QR | ATAGAAACCGACCTGGAT |
| H4QF | ACAACCTCCGAGTGATTT |
| 2436H | CAACATCGAGGTCGTAAA |
| 2684L | ACAGGGCTCGTTAGGGTGGC |
| 2914H | TTGAGGGCATCGGCAATA |
| 3326L | TCATCTCAACATTAGCCGAAAC |
| 3635L | TATGAAAGAACTTCCTACCAC |
| 4086L | CGAACCATAGAAGCCTCCAC |
| 4425L | GTAGGAGGCTGAGGCGGTCT |
| H4QR | GGATGATGCTGCTATTTT |
| H5QF | TCTAACCACTACACTAATCT |
| 5132H | TTCGTGGGATGGTAGCC |
| 5715H | TGTTCCGGCACCTGCTTCA |
| 5992L | CACAGACCGTAACCTGAACAC |
| 6092H | CGAAGCCTGGTAGGATAAG |
| H5QR | ATAAGTATCGTGGAGAAC |
| H6QF | TGCACGGAGGAAATATCAA |
| 6482L | CATTTCCATTATGTCCTG |
| 6542H | AGAATAGAGGGATCAGTGTA |
| 7419L | CCACATCAGACCTAAAACC |
| 7612L | CAGGACTTTACTACGGTCAATG |
| 7623H | TCTGAGCATTGACCGTAG |
| H6QR | ATTCTCAAGGGGCAGAGTG |
| H7B1F | TGCGGATCAAACCACAGCT |
| 7939L | AACACTCTGCCCCTTGA |
| H7B1R | AGTGTTGCCCCTCCGATGA |
| H7B2F | TTCATTGGCTCAACAAACT |
| 8439L | GGCTAACCGCTAACATT |
| H7B2R | GGTGGGCTCAGGTGATAGA |
| H8B1F | CTCTATCACCTGAGCCCACCAT |
| 9321L | CACTTTACATCCAGCCACC |
| 9367H | TATCAAGCGGCTGCTTCGA |
| H8B1R | CGTTCGCAGGCAGCAAATAC |
| H8B2F | AGCATTCACAGTATCCCTCGTA |
| 10150H | CTAGTAGGGATAATCCTAGAGC |
| 10621L | AGGCAACCAAACAGAACG |
| 10641H | CTGCGTTCAGGCGTTCTGTTT |
| 10841L | TCACCTCTGACTCCCAAAAGC |
| 10932H | TTCCGTAGCCTCCTAGTTTTA |
| H8B2R | TTAGATCGGTTTGTCGCAAG |
| H9QF | GTTCCATCTGCTTGCGACAA |
| 11287H | AATAGTCATCAGGCTGCTATAAGG |
| 11502H | GGCCATGAGTGCGTTTT |
| 11494L | GTGGATCTAGAAACAGAAACTTA |
| 11746H | TTGTAGGTATTGAAGATTGATAA |
| 11947L | TTCACTCTGGACAAGAAACAA |
| H9QR | TGCCTACTCCCTCCCATCC |
| H10B1F | TAACCGCATCGGGGATATC |
| 12537L | TAGTTGTAGCAGGCGTCTTCC |
| 12656H | CGCAGATTGCTGTGAATAGTG |
| 12819L | TTATCCACAGCCTAAATGACG |
| 13162H | GCCAATTAAGAGGCGTTTG |
| 13418H | GGCTTATTGATAGGTTAGCG |
| 13386L | GACTCCCACCGCTCGCTAA |
| 13644L | AGTCACAATCACAACCCAAGC |
| H10B1R | GGGCTGGTAGGTCAATAAAAGAG |
| H10B2F | AGTCACAATCACAACCCAAGC |
| 13644L | AGTCACAATCACAACCCAAGC |
| 14272L | GATGAAACTTCGGCTCCCT |
| 14355L | ATCAGACACGACAACTGCCTTCT |
| 14375H | AAGGCAGTTGTCGTGTCTGATGT |
| 14750L | TCATCACAGCCCTGGTAGTCG |
| 15223H | GGTGTTCCACTGGCTGTCC |
| H10B2R | GACCAGGGTAATGTGCGATAT |
|  | | | | |
| HDF | AGTCTCACCATCAACACCCAAAGC | r polymerase(5U/μl): 0.5μl  r buffer(+Mg^2+^, 10X): 5μl  dNTP Mix(2.5mM): 3μl  Primer F(10ρmol/μl): 1μl  Primer R(10ρmol/μl): 1μl  DNA(50ng/μl): 1μl  ddH_2_O: 38.5μl | Step1: 95°C 3min  Step2(35cycles): 94°C 50sec, 58°C 1min, 72°C 1min  Step3: 72°C 10min | 1) Blood and tissue samples;  2) HVSI PCR* |
| HDR | ACTCATCTAGGCATTTTCAGTG-3 |
|  | | | |  |
| HF | CCTGAAGTAGGAACCAGATG |  |  | 1) Blood and tissue samples;  2) HVSI Sequencing * |
| HDF | AGTCTCACCATCAACACCCAAAGC |
|  | | | |  |
| HCF3 | ATTTTTATACCACTCGCAAGCA | Phusion Taq(5U/μl): 0.5μl  Phusion buffer(10X): 10μl  dNTP Mix(2mM): 5μl  Primer F(5ρmol/μl): 5μl  Primer R(5ρmol/μl): 5μl  DNA: 1μl(50ng/μl) or 1disk  ddH_2_O: 23.5μl | Step1: 95°C 3min  Step2(35cycles): 98°C 5sec, 55°C 30sec, 72°C 30sec  Step3: 72°C 10min | 1) Hair root and buccal swap samples;  2) HVSI PCR |
| HCR3 | TATAAAAAGATACCAAATGCATGA |
| HCF2 | ATGAAGAGTCCCTGTAGTATATC |
| HCR2 | TTATGTGTGAGCATGGGCTGAT |
|  | | | |  |
| HCF1 | ATTACCCTGGTCTTGTAAACCA |  |  | 1) Hair root and buccal swap samples;2) HVSI Sequencing |
| HCR1 | TCATTAGTCCATCGAGATGTCT |

Note: 5ml 10X reaction buffer, 1.5mM MgCl_2_, 200μM dNTPs, 10pM each primer, 1U Taq DNA polymerase (TaKaRa Biosystems).

*references [29]
