# Supplementary material for: Local origin or external input: modern horse origin in East Asia
Source: BMC Evol Biol. 2019 Nov 27;19:217. doi: 10.1186/s12862-019-1532-y (PMC6882189; doi:10.1186/s12862-019-1532-y)
Supplement: Supplementary file 5 — Additional file 5: Table S5. A comparison between the new and the old nomenclatures for horse mtDNA haplogroups. [file 12862_2019_1532_MOESM5_ESM.doc]

**Additional file 5: Table S5** A comparison between the new and the old nomenclatures for horse mtDNA haplogroups

| References  [9, 10, 49] | References  [5] | References  [42] | This study |
| --- | --- | --- | --- |
| A4, A5 | D2 | A | A |
| A3 | D3 | B | B |
|  |  | C | C |
| E | G | D | D |
|  |  | E | E |
| A2 |  | F | F |
| A1 | X3c | G | G |
|  | X4 | H | H |
| B1, B2 | I | I | I |
|  |  | J | JK |
|  |  | K | JK |
| D1,D2,D3 | X1,X2 | L | L |
| C1 | B | M | M |
| C2 | F | N | N |
| F1 | K3 | O | OP |
| F1 | K3 | P | OP |
| F2,F3 | K2 | Q | Q |
| G | X7 | R | R |

* Nomenclature by the following references [5, 9, 10, 42,49]
