# Supplementary material for: Local origin or external input: modern horse origin in East Asia
Source: BMC Evol Biol. 2019 Nov 27;19:217. doi: 10.1186/s12862-019-1532-y (PMC6882189; doi:10.1186/s12862-019-1532-y)
Supplement: Supplementary file 6 — Additional file 6: Table S6. Results of BMCMC (Bayesian Markov Chain Monte Carlo) with RLGC-S (relaxed lognormal clock rate and population size estimated from Bayesian skyline plot) model. [file 12862_2019_1532_MOESM6_ESM.doc]

**Additional file 6: Table S6** Results of BMCMC (Bayesian Markov Chain Monte Carlo) with RLGC-S (relaxed lognormal clock rate and population size estimated from Bayesian skyline plot) model.

|  | Mean | Stdev |
| --- | --- | --- |
| tmrca(A) | 0.3225 | 7.9818E-3 |
| Tmrca(A-B) | 0.695 | 1.6179E-2 |
| Tmrca(A-C) | 1.0961 | 2.4644E-2 |
| Tmrca(A-D) | 1.5076 | 3.3824E-2 |
| Tmrca(A-G) | 2.0317 | 4.7258E-2 |
| Tmrca(A-H) | 2.0324 | 4.7284E-2 |
| Tmrca(A-I) | 2.0215 | 4.6971E-2 |
| Tmrca(A-K) | 2.8083 | 6.4471E-2 |
| Tmrca(A-L) | 3.0806 | 6.9595E-2 |
| Tmrca(A-Q) | 3.8368 | 5.8979E-2 |
| Tmrca(B) | 0.4282 | 1.0405E-2 |
| Tmrca(C) | 0.3431 | 8.6252E-3 |
| Tmrca(D) | 0.5965 | 1.3905E-2 |
| Tmrca(E) | 0.2299 | 6.1141E-3 |
| Tmrca(E-G) | 1.1971 | 2.9144E-2 |
| Tmrca(F) | 0.1914 | 5.0617E-3 |
| Tmrca(G) | 0.4837 | 1.2546E-2 |
| Tmrca(H) | 0.5039 | 1.2324E-2 |
| Tmrca(I) | 1.0097 | 2.7496E-2 |
| Tmrca(JK) | 1.6824 | 4.0285E-2 |
| Tmrca(L) | 0.9011 | 2.2579E-2 |
| Tmrca(M) | 0.3463 | 8.2712E-3 |
| Tmrca(M-N) | 0.9291 | 2.2706E-2 |
| Tmrca(M-Q) | 2.5607 | 4.0258E-2 |
| Tmrca(N) | 0.4341 | 1.091E-2 |
| Tmrca(O-Q) | 1.0007 | 2.2896E-2 |
| Tmrca(OP) | 0.5447 | 1.3129E-2 |
| Tmrca(Q) | 0.4248 | 1.0887E-2 |
| Tmrca(R) | 0.6448 | 1.8381E-2 |
| Tmrca(A-R) | 4.2897 | 1.3698E-3 |

Mean: the mean value of the sampled trace across the chain;

Stdev: the standard deviation of the mean
