# Supplementary material for: Local origin or external input: modern horse origin in East Asia
Source: BMC Evol Biol. 2019 Nov 27;19:217. doi: 10.1186/s12862-019-1532-y (PMC6882189; doi:10.1186/s12862-019-1532-y)
Supplement: Supplementary file 7 — Additional file 7: Table S7. Matrix of the gene flows (Nm) (below diagonal) among 14 populations of the domestic horse across the world. [file 12862_2019_1532_MOESM7_ESM.doc]

Additional file 7: Table S7 Matrix of the gene flows (*N*_m_) (below diagonal) among 14 populations of domestic horse across world.

| **Population** | AF | CA | CE | NEA | SEA | EE | NA | NAM | NE | SA | SAM | SE | WA | WE |
| --- | --- | --- | --- | --- | --- | --- | --- | --- | --- | --- | --- | --- | --- | --- |
| AF |  |  |  |  |  |  |  |  |  |  |  |  |  |  |
| CA | 31.557 |  |  |  |  |  |  |  |  |  |  |  |  |  |
| CE | 67.986 | 11.295 |  |  |  |  |  |  |  |  |  |  |  |  |
| NEA | 4.246 | 2.235 | 7.763 |  |  |  |  |  |  |  |  |  |  |  |
| SEA | 10.933 | 4.997 | 29.501 | 23.014 |  |  |  |  |  |  |  |  |  |  |
| EE | 2.495 | 1.437 | 4.123 | 81.224 | 8.771 |  |  |  |  |  |  |  |  |  |
| NA | 6.7818 | 3.035 | 11.039 | 38.763 | 24.311 | 15.103 |  |  |  |  |  |  |  |  |
| NAM | 2.976 | 1.661 | 4.643 | 59.613 | 8.888 | 25.780 | 18.889 |  |  |  |  |  |  |  |
| NE | 4.786 | 2.266 | 10.802 | ∞ | 31.205 | ∞ | 32.815 | 12.284 |  |  |  |  |  |  |
| SA | 16.639 | 6.783 | 52.797 | 18.336 | 53.707 | 8.0626 | 25.026 | 7.746 | 51.791 |  |  |  |  |  |
| SAM | 7.496 | 3.413 | 16.242 | 186.182 | 49.583 | 19.156 | 135.185 | 18.926 | ∞ | 43.198 |  |  |  |  |
| SE | 14.541 | 6.078 | 80.81 | 12.644 | 25.341 | 6.233 | 15.129 | 7.710 | 18.072 | 23.943 | 20.868 |  |  |  |
| WA | 4.068 | 2.231 | 6.0326 | 144.94 | 12.052 | 23.638 | 34.781 | 34.665 | 21.205 | 10.456 | 41.010 | 7.834 |  |  |
| WE | 5.6197 | 2.911 | 9.6973 | 40.223 | 17.951 | 11.734 | 71.043 | 20.666 | 17.078 | 13.112 | 37.929 | 16.303 | 26.283 |  |
