# Supplementary material for: Local origin or external input: modern horse origin in East Asia
Source: BMC Evol Biol. 2019 Nov 27;19:217. doi: 10.1186/s12862-019-1532-y (PMC6882189; doi:10.1186/s12862-019-1532-y)
Supplement: Supplementary file 8 — Additional file 8: Table S8. Dominant haplotypes distribution of haplogroups in horse populations. [file 12862_2019_1532_MOESM8_ESM.doc]

Additional file 8: Table S8 Dominant haplotypes distribution of haplogroups in horse populations

| haplogroup | dH | haplotype | Sample no. of dH | AF  (46) | CA  (61) | WA  (251) | EE  (31) | NE  (81) | CE  (163) | SE  (488) | WE  (520) | SA  (16) | SEA  (923) | NEA  (718) | NA  (68) | NAM  (115) | SAM  (73) |
| --- | --- | --- | --- | --- | --- | --- | --- | --- | --- | --- | --- | --- | --- | --- | --- | --- | --- |
| D | HapD2 | Hap2 | 15 | 0 | 3 | 0 | 1 | 1 | 0 | 2 | 5 | 0 | 1 | 2 | 0 | 0 | 0 |
|  | HapD4 | Hap296 | 23 | 0 | 0 | 1 | 0 | 0 | 0 | 0 | 0 | 1 | 11 | 10 | 0 | 0 | 0 |
|  | HapD7 | Hap10 | 35 | 0 | 0 | 0 | 2 | 1 | 0 | 0 | 30 | 0 | 0 | 2 | 0 | 0 | 0 |
|  | HapD10 | Hap97 | 34 | 0 | 0 | 0 | 0 | 0 | 1 | 2 | 3 | 0 | 16 | 12 | 0 | 0 | 0 |
|  | Td | | 107 | 0 | 3 | 1 | 3 | 2 | 1 | 4 | 38 | 1 | 28 | 26 | 0 | 0 | 0 |
|  | T | | 144 | 0 | 6 | 2 | 3 | 6 | 1 | 4 | 48 | 1 | 44 | 29 | 0 | 0 | 0 |
| EFG | HapEFG2 | Hap4 | 75 | 1 | 6 | 2 | 4 | 0 | 2 | 16 | 7 | 0 | 5 | 24 | 2 | 6 | 0 |
|  | HapEFG6 | Hap24 | 97 | 0 | 2 | 7 | 0 | 1 | 6 | 26 | 1 | 1 | 26 | 29 | 1 | 0 | 1 |
|  | HapEFG8 | Hap121 | 27 | 0 | 0 | 1 | 0 | 0 | 0 | 14 | 0 | 1 | 5 | 4 | 2 | 0 | 0 |
|  | Td | | 199 | 1 | 8 | 10 | 4 | 1 | 8 | 56 | 8 | 2 | 36 | 57 | 5 | 6 | 1 |
|  | T |  | 323 | 1 | 9 | 24 | 4 | 5 | 14 | 75 | 30 | 2 | 70 | 72 | 8 | 6 | 3 |
| H | HapH1 | Hap51 | 31 | 0 | 0 | 1 | 0 | 0 | 0 | 7 | 8 | 0 | 4 | 9 | 1 | 0 | 0 |
|  | HapH2 | Hap107 | 17 | 0 | 0 | 0 | 0 | 0 | 1 | 1 | 0 | 0 | 14 | 1 | 0 | 0 | 0 |
|  | Td | | 48 | 0 | 0 | 1 | 0 | 0 | 1 | 8 | 8 | 0 | 18 | 10 | 1 | 0 | 0 |
|  | T | | 59 | 0 | 0 | 1 | 0 | 0 | 1 | 9 | 9 | 1 | 26 | 11 | 1 | 0 | 0 |
| I | HapI3 | Hap213 | 37 | 0 | 2 | 6 | 0 | 0 | 1 | 0 | 1 | 1 | 11 | 15 | 0 | 0 | 0 |
|  | HapI7 | Hap46 | 20 | 0 | 0 | 2 | 0 | 1 | 0 | 4 | 11 | 0 | 0 | 2 | 0 | 0 | 0 |
|  | HapI10 | Hap49 | 21 | 0 | 0 | 0 | 0 | 1 | 0 | 0 | 19 | 0 | 0 | 1 | 0 | 0 | 0 |
|  | HapI13 | Hap80 | 53 | 0 | 1 | 6 | 0 | 0 | 10 | 13 | 9 | 1 | 0 | 7 | 4 | 0 | 3 |
|  | Td | | 131 | 0 | 3 | 14 | 0 | 2 | 11 | 17 | 40 | 2 | 11 | 25 | 4 | 0 | 3 |
|  | T | | 199 | 1 | 4 | 23 | 6 | 5 | 12 | 26 | 61 | 4 | 15 | 33 | 4 | 0 | 5 |
| L | HapL1 | Hap39 | 203 | 3 | 1 | 19 | 0 | 2 | 12 | 20 | 44 | 0 | 63 | 26 | 5 | 4 | 4 |
|  | HapL3 | Hap26 | 156 | 0 | 6 | 12 | 0 | 2 | 12 | 15 | 34 | 1 | 48 | 17 | 0 | 6 | 3 |
|  | HapL4 | Hap53 | 215 | 13 | 3 | 12 | 0 | 0 | 9 | 64 | 28 | 2 | 3 | 22 | 1 | 45 | 13 |
|  | HapL9 | Hap31 | 99 | 1 | 0 | 5 | 0 | 2 | 4 | 21 | 7 | 0 | 48 | 7 | 0 | 0 | 4 |
|  | Td | | 673 | 14 | 10 | 48 | 0 | 4 | 37 | 120 | 113 | 3 | 162 | 72 | 6 | 55 | 24 |
|  | T | | 884 | 22 | 10 | 57 | 2 | 6 | 52 | 163 | 178 | 3 | 196 | 81 | 13 | 71 | 30 |
| M | HapM1 | Hap13 | 146 | 0 | 5 | 10 | 2 | 10 | 6 | 5 | 27 | 0 | 52 | 25 | 1 | 3 | 0 |
|  | HapM4 | Hap16 | 39 | 0 | 1 | 0 | 1 | 1 | 0 | 4 | 22 | 0 | 7 | 3 | 0 | 0 | 0 |
|  | Td | | 185 | 0 | 6 | 10 | 3 | 11 | 6 | 9 | 49 | 0 | 59 | 28 | 1 | 3 | 0 |
|  | T | | 208 | 0 | 7 | 11 | 3 | 12 | 6 | 11 | 59 | 0 | 63 | 28 | 1 | 3 | 4 |
| N | HapN1 | Hap14 | 87 | 0 | 0 | 10 | 1 | 4 | 5 | 23 | 17 | 0 | 0 | 19 | 0 | 2 | 7 |
|  | HapN4 | Hap35 | 28 | 0 | 0 | 2 | 0 | 3 | 1 | 3 | 11 | 0 | 0 | 5 | 0 | 0 | 2 |
|  | Td | | 115 | 0 | 0 | 12 | 1 | 7 | 6 | 26 | 28 | 0 | 0 | 24 | 0 | 2 | 9 |
|  | T | | 157 | 0 | 0 | 12 | 3 | 7 | 9 | 39 | 36 | 0 | 3 | 33 | 1 | 5 | 9 |
| OP | HapOP3 | Hap7 | 40 | 1 | 0 | 2 | 2 | 0 | 1 | 0 | 0 | 0 | 21 | 14 | 0 | 0 | 0 |
|  | HapOP4 | Hap23 | 57 | 0 | 1 | 7 | 0 | 2 | 7 | 2 | 0 | 0 | 12 | 24 | 1 | 0 | 1 |
|  | Td | | 97 | 1 | 1 | 9 | 2 | 2 | 8 | 2 | 0 | 0 | 33 | 38 | 1 | 0 | 1 |
|  | T | | 160 | 1 | 1 | 17 | 2 | 3 | 9 | 9 | 0 | 0 | 44 | 67 | 6 | 0 | 1 |
| Q | HapQ1 | Hap30 | 39 | 0 | 1 | 1 | 0 | 5 | 2 | 5 | 12 | 0 | 4 | 9 | 0 | 0 | 0 |
|  | HapQ2 | Hap165 | 31 | 0 | 0 | 0 | 0 | 0 | 0 | 1 | 0 | 0 | 12 | 16 | 1 | 0 | 1 |
|  | HapQ6 | Hap94 | 29 | 0 | 0 | 6 | 0 | 0 | 1 | 3 | 0 | 0 | 3 | 13 | 0 | 0 | 0 |
|  | HapQ9 | Hap123 | 56 | 0 | 2 | 0 | 0 | 0 | 0 | 3 | 0 | 0 | 14 | 33 | 2 | 1 | 1 |
|  | Td | | 155 | 0 | 3 | 7 | 0 | 5 | 3 | 14 | 12 | 0 | 33 | 71 | 3 | 1 | 2 |
|  | T | | 275 | 0 | 10 | 21 | 0 | 7 | 4 | 22 | 14 | 1 | 87 | 103 | 3 | 1 | 2 |
| R | HapR1 | Hap56 | 14 | 0 | 1 | 1 | 0 | 0 | 0 | 6 | 1 | 0 | 3 | 2 | 0 | 0 | 0 |
|  | HapR2 | Hap169 | 51 | 0 | 0 | 1 | 0 | 0 | 20 | 1 | 0 | 0 | 22 | 5 | 1 | 0 | 0 |
|  | Td | | 65 | 0 | 1 | 2 | 0 | 0 | 20 | 7 | 1 | 0 | 25 | 7 | 1 | 0 | 0 |
|  | T | | 79 | 0 | 1 | 2 | 0 | 0 | 21 | 8 | 1 | 0 | 34 | 11 | 1 | 0 | 0 |

Number in the bracket is the sample size of each population; dH: dominant haplotype; T: total sample size of each haplogroup and sample distribution; Td: total sample size of these dominant haplotypes and sample distribution
