# Supplementary material for: Local origin or external input: modern horse origin in East Asia
Source: BMC Evol Biol. 2019 Nov 27;19:217. doi: 10.1186/s12862-019-1532-y (PMC6882189; doi:10.1186/s12862-019-1532-y)
Supplement: Supplementary file 9 — Additional file 9: Table S9. Genetic statistics for haplogroups in Europe, Central-West Asia, and East Asia modern horse populations 100,000 resampling. [file 12862_2019_1532_MOESM9_ESM.doc]

**Additional file 9: Table S9 Genetic statistics for haplogroups in Europe, Central-West Asia and East Asia modern horse populations 100000 resampling**

|  | Europe | | | | | | Central and West Asia | | | | | | East Asia | | | | | |
| --- | --- | --- | --- | --- | --- | --- | --- | --- | --- | --- | --- | --- | --- | --- | --- | --- | --- | --- |
|  | N | H | UH | Hres | F% | π | N | H | UH | Hres | F% | π | N | H | UH | Hres | F% | π |
| hapD | 62 | 15 | 9 | 4.10±1.27 | 60% | 0.0046±0.0033 | 8 | 6 | 5 | 6.00±0 | 83.3% | 0.0093±0.0064 | 62 | 14 | 7 | 4.38±1.28 | 50% | 0.0063±0.0042 |
| hapEFG | 128 | 27 | 19 | 12.68±3.60 | 70.4% | 0.0083±0.0052 | 33 | 11 | 4 | 11.00±0 | 36.4% | 0.0074±0.0049 | 142 | 23 | 15 | 10.53±2.94 | 65.2% | 0.0082±0.0051 |
| HapH | 19 | 4 | 2 | — | 50% | 0.0017±0.0018 | 1 | 1 | 0 | 1.00±0 | 0 | 0 | 37 | 5 | 3 | — | 60% | 0.0037±0.0029 |
| hapI | 110 | 19 | 12 | 9.79±2.48 | 63.2% | 0.0074±0.0048 | 27 | 8 | 1 | 8.00±0 | 20% | 0.0084±0.0054 | 48 | 13 | 7 | 9.05±1.83 | 53.8% | 0.0055±0.0038 |
| hapL | 401 | 51 | 39 | 17.45±6.16 | 76.5% | 0.0075±0.0048 | 67 | 11 | 3 | 11.00±0 | 27.27% | 0.0062±0.0041 | 277 | 28 | 16 | 12.01±4.13 | 57.14% | 0.0057±0.0039 |
| HapM | 91 | 8 | 6 | 3.95±0.94 | 75% | 0.0030±0.0025 | 18 | 3 | 1 | 3.00±0 | 33.3% | 0.0013±0.0015 | 91 | 6 | 4 | 2.70±0.68 | 66.7% | 0.0012±0.0014 |
| hapN | 101 | 22 | 18 | 4.95±1.86 | 81.8% | 0.0050±0.0035 | 12 | 2 | 0 | 2.00±0 | 0 | 0.0013±0.0015 | 36 | 7 | 3 | 4.88±0.89 | 42.9% | 0.0045±0.0033 |
| hapOP | 23 | 8 | 3 | 7.22±0.52 | 37.5% | 0.0055±0.0039 | 18 | 8 | 3 | 8.00±0 | 37.5% | 0.0051±0.0037 | 111 | 20 | 14 | 7.31±2.30 | 70% | 0.0062±0.0042 |
| hapQ | 47 | 16 | 3 | 12.22±1.90 | 18.75% | 0.0053±0.0037 | 31 | 14 | 4 | 14.00±0 | 28.57% | 0.0085±0.0054 | 190 | 34 | 18 | 13.49±3.53 | 52.94% | 0.0080±0.0050 |
| hapR | 30 | 4 | 1 | 1.75±0.35 | 25% | 0.0020±0.0020 | 3 | 2 | 0 | 2.00±0 | 0 | 0.0027±0.0034 | 45 | 11 | 8 | 2.05±0.50 | 72.7% | 0.0035±0.0028 |

N - number of individuals

H - nunber of haplotypes.

UH - number of unique haplotypes.

Hres - number of haplotypes (with SD) obtained from resampling of the least size among the three populations (100000 replications) for each haplogroups to adjust for different sample size.

F% - the ratio of the number of unique haplotype to the number of haplotype.

π - nucleotide diversity (with SD).

—can not be calculated
