# Supplementary material for: Local origin or external input: modern horse origin in East Asia
Source: BMC Evol Biol. 2019 Nov 27;19:217. doi: 10.1186/s12862-019-1532-y (PMC6882189; doi:10.1186/s12862-019-1532-y)
Supplement: Supplementary file 10 — Additional file 10: Table S10. Sample size and haplogroup frequency distribution of domestic horse in East Asia. [file 12862_2019_1532_MOESM10_ESM.doc]

**Additional file 10: Table S10**. Sample size and haplogroup frequency distribution of domestic horse in East Asia

| Population | Sample size | Haplogroup frequency distribution | | | | | | | | | |
| --- | --- | --- | --- | --- | --- | --- | --- | --- | --- | --- | --- |
|  |  | hap D | hap EFG | hap H | hap I | hap L | hap M | hap N | hap OP | hap Q | hap R |
| NEA | 509 | 7.07% | 14.73% | 2.16% | 6.48% | 19.06% | 5.5% | 6.29% | 13.16% | 23.38% | 2.16% |
| SEA | 551 | 6.72% | 13.07% | 5.44% | 2.54% | 32.49% | 11.43% | 0.73% | 7.99% | 13.07% | 6.53% |
