# Supplementary material for: Local origin or external input: modern horse origin in East Asia
Source: BMC Evol Biol. 2019 Nov 27;19:217. doi: 10.1186/s12862-019-1532-y (PMC6882189; doi:10.1186/s12862-019-1532-y)
Supplement: Supplementary file 11 — Additional file 11: Table S11. Genetic diversity for control-region sequences of the modern domestic horse across the world (DOC 44 kb) [file 12862_2019_1532_MOESM11_ESM.doc]

**Additional file 11: Table S11.** Genetic diversity for control-region sequences of modern domestic horse across the world.

| Region^a^ | N | Hn | UH | π | H | UT |
| --- | --- | --- | --- | --- | --- | --- |
| AF | 46 | 21 | 9 | 0.0220 ± 0.0120 | 0.8947 ± 0.0330 | 60.7% |
| CA | 61 | 30 | 4 | 0.0257 ± 0.0137 | 0.9650 ± 0.0092 | 68.9% |
| CE | 163 | 56 | 14 | 0.0291 ± 0.0152 | 0.9583 ± 0.0061 | 44.8% |
| NEA | 718 | 159 | 70 | 0.0256 ± 0.0134 | 0.9782 ± 0.0015 | 39.3% |
| SEA | 923 | 146 | 80 | 0.0249 ± 0.0131 | 0.9502 ± 0.0036 | 45.1% |
| EE | 31 | 17 | 2 | 0.0242 ± 0.0132 | 0.9398 ± 0.0248 | 38.7% |
| NA | 68 | 35 | 10 | 0.0249 ± 0.0133 | 0.9622 ± 0.0116 | 41.2% |
| NAM | 115 | 20 | 3 | 0.0208 ± 0.0113 | 0.7945 ± 0.0296 | 79.1% |
| NE | 81 | 40 | 10 | 0.0208 ± 0.0113 | 0.9611 ± 0.0099 | 32.1% |
| SA | 16 | 15 | 2 | 0.0256 ± 0.0144 | 0.9917 ± 0.0254 | 31.2% |
| SAM | 73 | 36 | 13 | 0.0254 ± 0.0135 | 0.9505 ± 0.0145 | 50.7% |
| SE | 488 | 144 | 71 | 0.0278 ± 0.0145 | 0.9648 ± 0.0038 | 48.8% |
| WA | 251 | 86 | 22 | 0.0260 ± 0.0137 | 0.9767 ± 0.0027 | 42.2% |
| WE | 520 | 100 | 50 | 0.0268 ± 0.0140 | 0.9680 ± 0.0022 | 35.2% |

^a^Region –see supplementary table 8 in detail.

N - number of individuals from geographic regions

Hn - nunber of haplotypes

UH - number of unique haplotypes

π - Nucleotide diversity

H – haplotype diversity

UT: the proportion of individuals having 12 universally occurring haplotypes (hap3,4,13,14,15,23,24,26,31,39,41,53).
