# Supplementary figures and images for: Local origin or external input: modern horse origin in East Asia
Source: BMC Evol Biol. 2019 Nov 27;19:217. doi: 10.1186/s12862-019-1532-y (PMC6882189; doi:10.1186/s12862-019-1532-y)

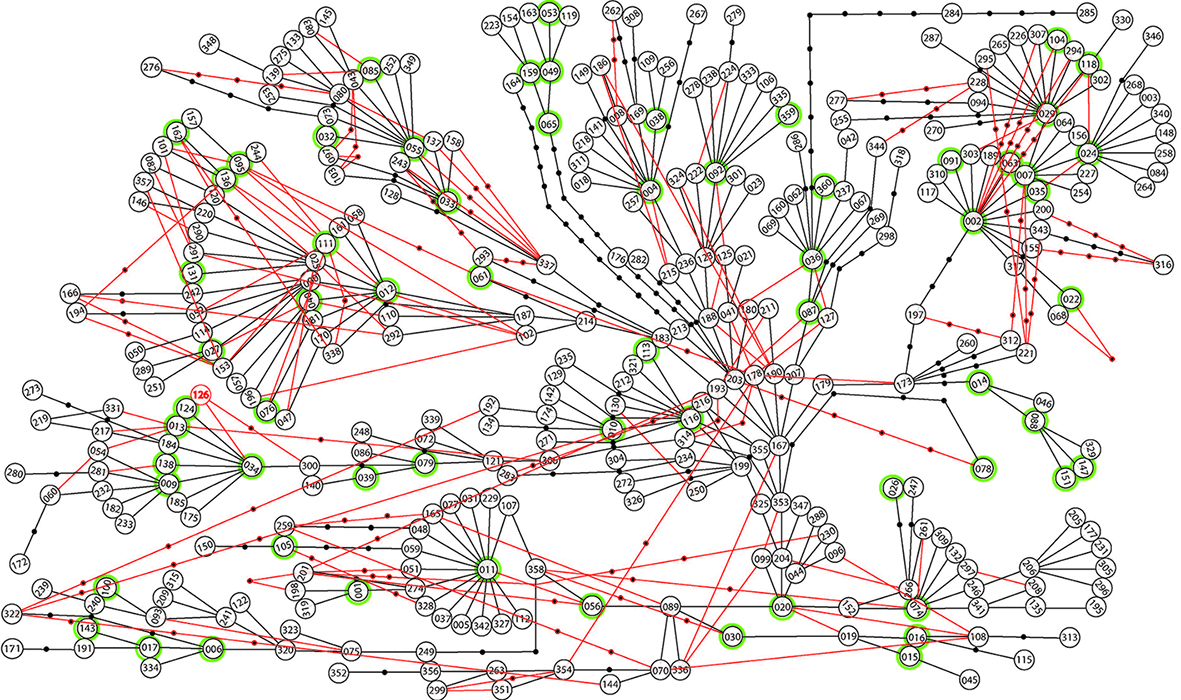

Supplement: Supplementary file 12 — Additional file 12: Figure S1. The choice criterion for sequencing mitochondrial genome sequence in this study. [file 12862_2019_1532_MOESM12_ESM.jpg]

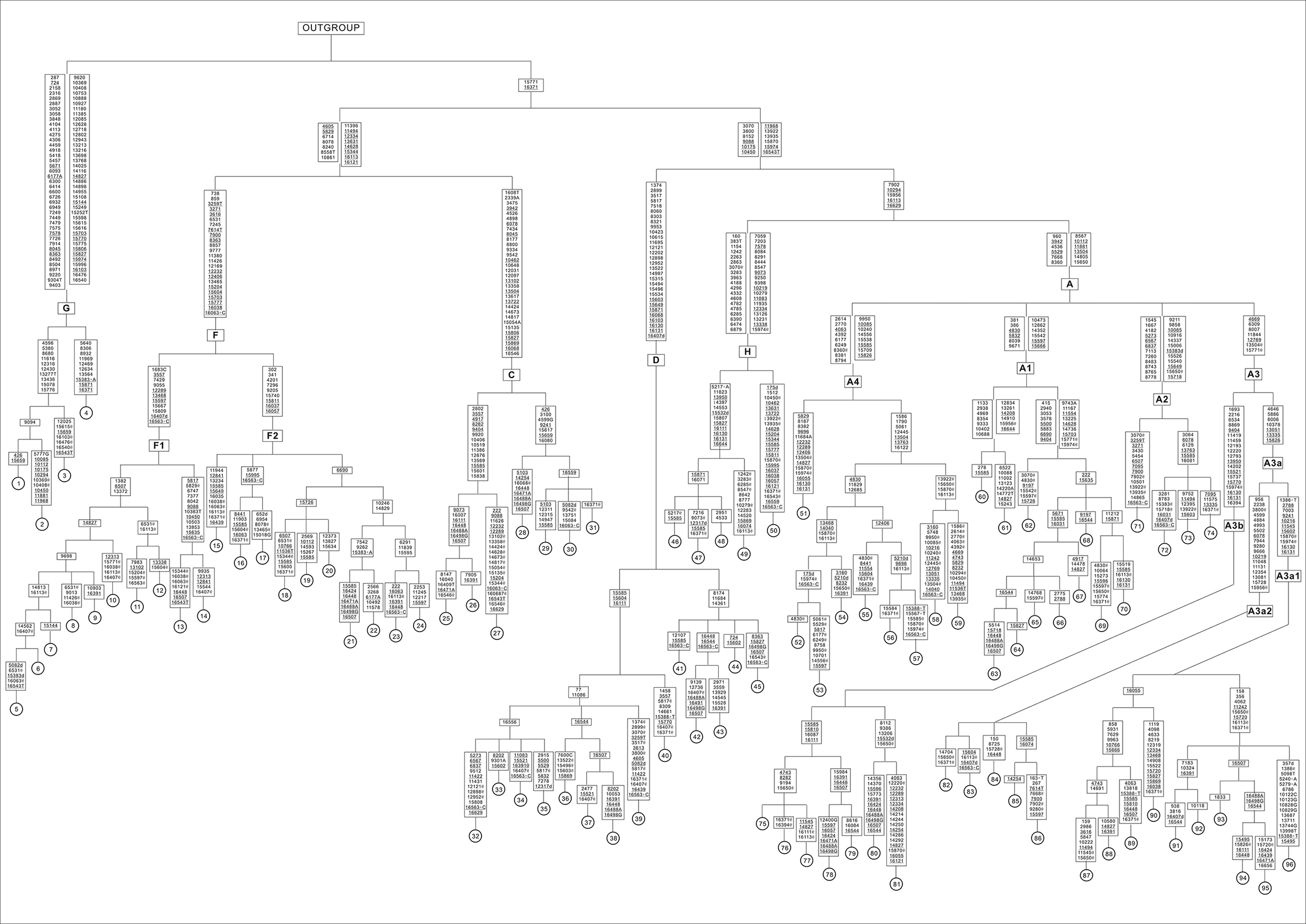

Supplement: Supplementary file 13 — Additional file 13: Figure S2. Classification tree of complete mtDNAs analyzed in this study. Suffixes A, C, G, and T indicate transversions, d indicates deletion; recurrent mutations are underlined; + indicates insertion. The prefix h indicates heteroplasmy and @ highlights back mutation. Numbers in circles are consistent with Additional file 4: Table S4. [file 12862_2019_1532_MOESM13_ESM.jpg]

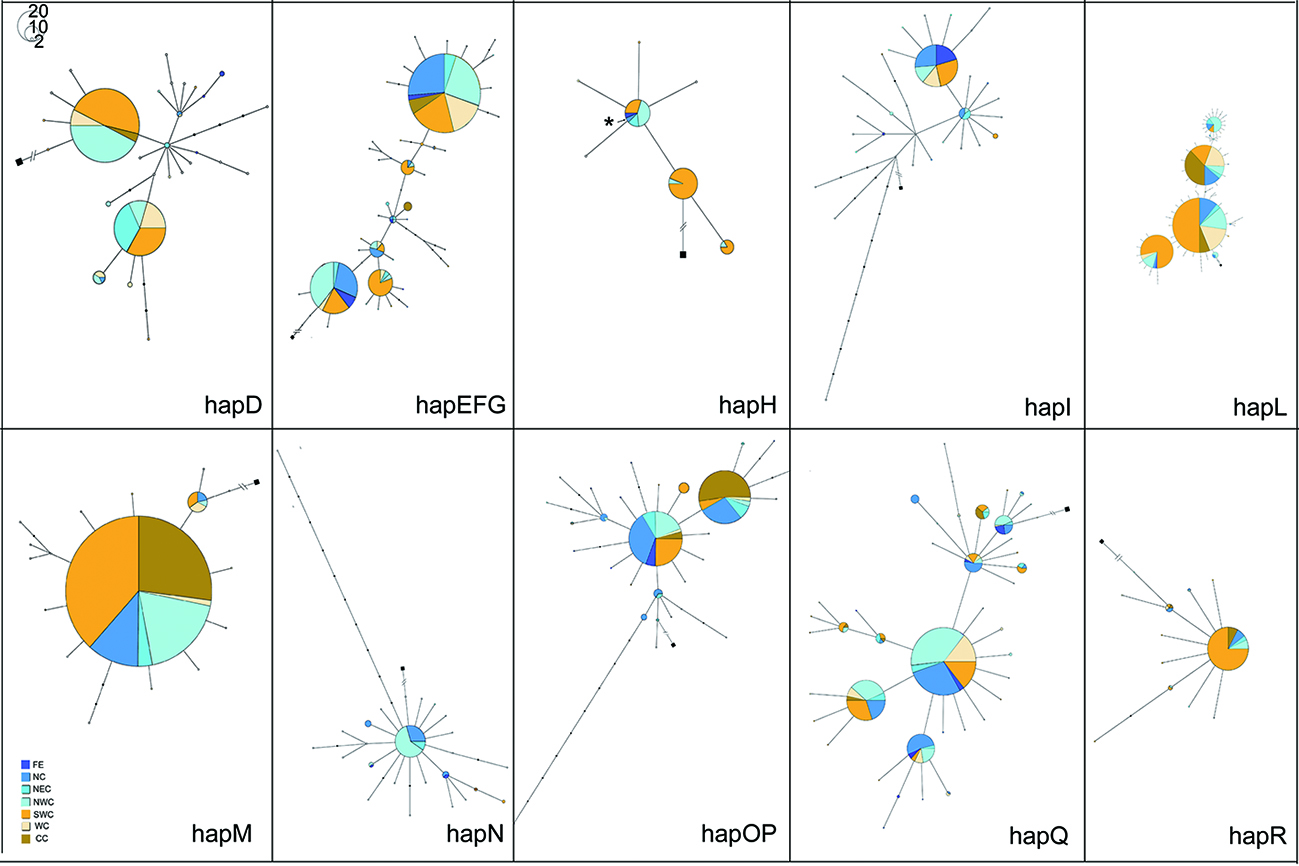

Supplement: Supplementary file 14 — Additional file 14: Figure S3. Minimum spanning network of haplogroups in East Asia. [file 12862_2019_1532_MOESM14_ESM.jpg]

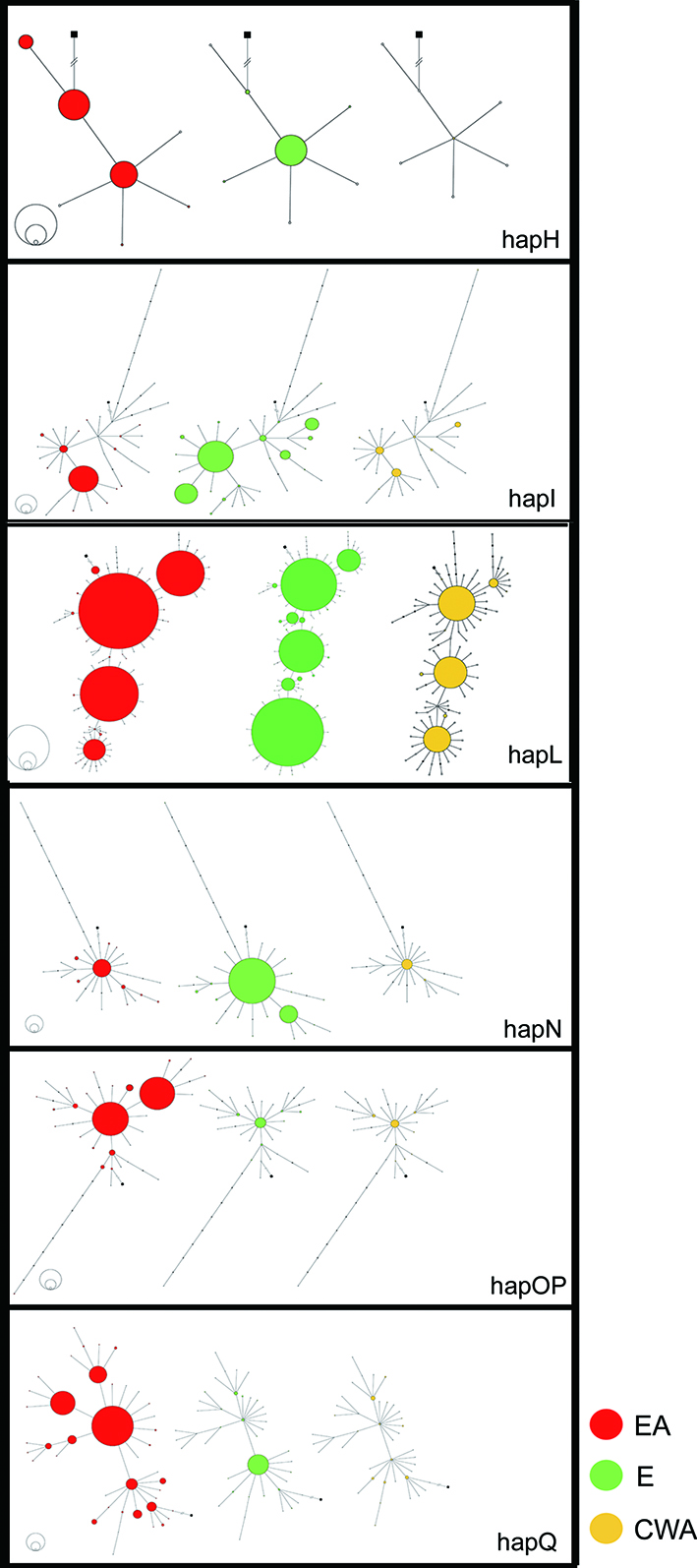

Supplement: Supplementary file 15 — Additional file 15: Figure S4. Haplotype frequency in three putative horse domestication regions (Europe, Central and West Asia, East Asia). [file 12862_2019_1532_MOESM15_ESM.jpg]

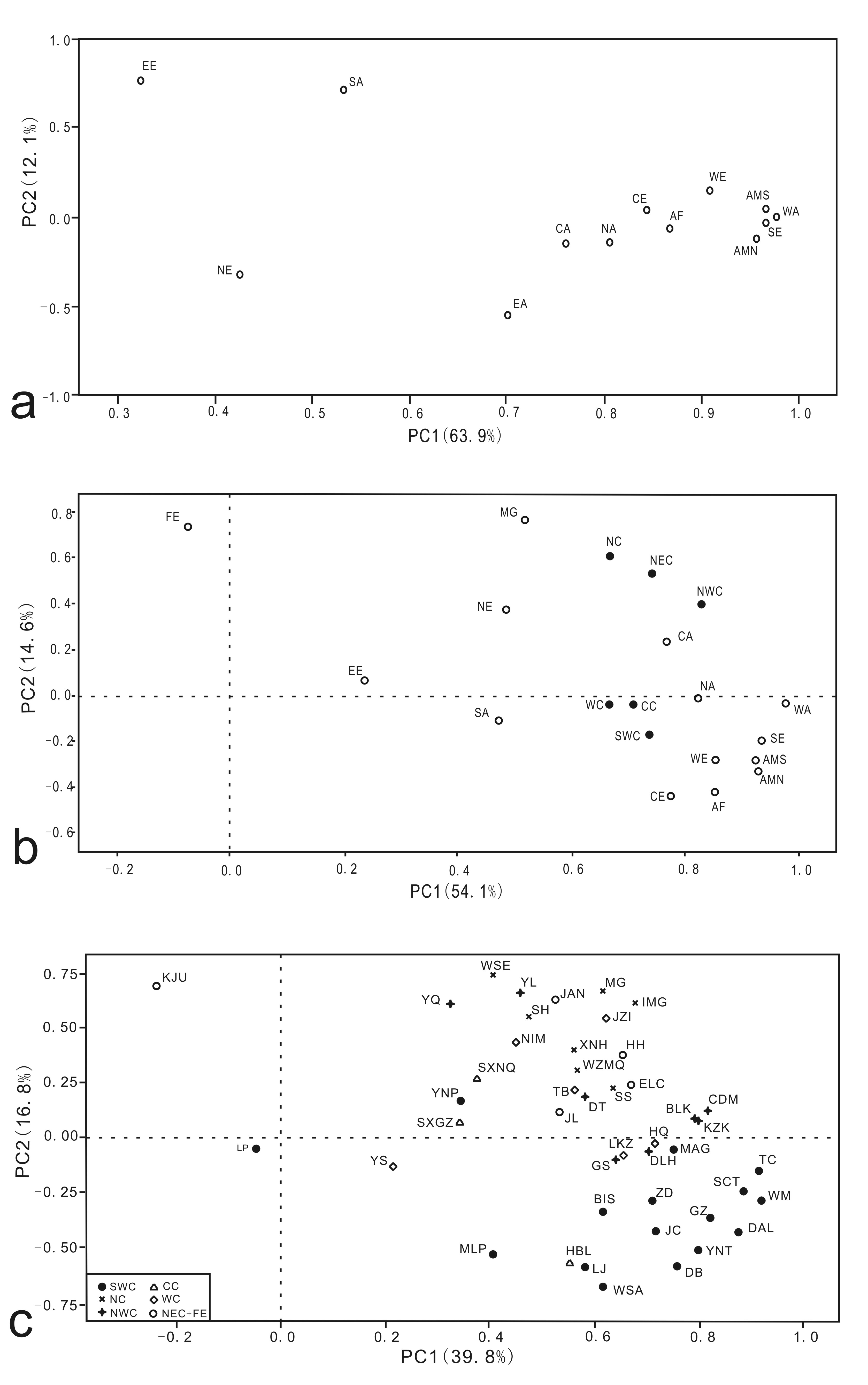

Supplement: Supplementary file 16 — Additional file 16: Figure S5. Region-based PC analysis of mtDNA haplogroup profiles in East Asia. (a) PC map of the 13 world horse populations (EE, NE, WE, SE, CE, CA, WA, NA, EA, SA, AF, NAM, SAM) based on haplogroup frequencies. (b) PC map of the 18 world horse populations based on haplogroup frequencies. EA is divided into subpopulations NC, NWC, NEC, SWC, CC, WC. (b) PC map of the East Asia horse populations based on haplogroup frequencies. For more details, see Table 1. [file 12862_2019_1532_MOESM16_ESM.jpg]
